# Risk factors associated with meiotic errors in blastocysts with mosaic results on biopsy

**DOI:** 10.1093/hropen/hoag067

**Published:** 2026-07-24

**Authors:** Jialiu Liu, Bing Cai, Yan Xu, Jing Wang, Xixiong Ai, Yanhong Zeng, Jing Guo, Rong Li, Jiafu Pan, Chenhui Ding, Yuanlin Ma, Fang Gu, Yi Zhou, Sijia Lu, Jieliang Ma, Cheng Wan, Jia Fei, Jiucheng Chen, Bingjie Lu, Haoting Zhang, Canquan Zhou, Yanwen Xu

**Affiliations:** Reproductive Medicine Center, The First Affiliated Hospital of Sun Yat-Sen University, Guangzhou, China; Key Laboratory for Reproductive Medicine of Guangdong Province, The First Affiliated Hospital of Sun Yat-Sen University, Guangzhou, China; Guangdong Provincial Clinical Research Center for Obstetrical and Gynecological Diseases, The First Affiliated Hospital of Sun Yat-Sen University, Guangzhou, China; Reproductive Medicine Center, The First Affiliated Hospital of Sun Yat-Sen University, Guangzhou, China; Key Laboratory for Reproductive Medicine of Guangdong Province, The First Affiliated Hospital of Sun Yat-Sen University, Guangzhou, China; Guangdong Provincial Clinical Research Center for Obstetrical and Gynecological Diseases, The First Affiliated Hospital of Sun Yat-Sen University, Guangzhou, China; Reproductive Medicine Center, The First Affiliated Hospital of Sun Yat-Sen University, Guangzhou, China; Key Laboratory for Reproductive Medicine of Guangdong Province, The First Affiliated Hospital of Sun Yat-Sen University, Guangzhou, China; Guangdong Provincial Clinical Research Center for Obstetrical and Gynecological Diseases, The First Affiliated Hospital of Sun Yat-Sen University, Guangzhou, China; Reproductive Medicine Center, The First Affiliated Hospital of Sun Yat-Sen University, Guangzhou, China; Key Laboratory for Reproductive Medicine of Guangdong Province, The First Affiliated Hospital of Sun Yat-Sen University, Guangzhou, China; Guangdong Provincial Clinical Research Center for Obstetrical and Gynecological Diseases, The First Affiliated Hospital of Sun Yat-Sen University, Guangzhou, China; Reproductive Medicine Center, The First Affiliated Hospital of Sun Yat-Sen University, Guangzhou, China; Department of Reproductive Medicine Center, Shenzhen Maternity and Child Healthcare Hospital, Women and Children's Medical Center, Southern Medical University, Shenzhen, China; Reproductive Medicine Center, The First Affiliated Hospital of Sun Yat-Sen University, Guangzhou, China; Key Laboratory for Reproductive Medicine of Guangdong Province, The First Affiliated Hospital of Sun Yat-Sen University, Guangzhou, China; Guangdong Provincial Clinical Research Center for Obstetrical and Gynecological Diseases, The First Affiliated Hospital of Sun Yat-Sen University, Guangzhou, China; Reproductive Medicine Center, The First Affiliated Hospital of Sun Yat-Sen University, Guangzhou, China; Key Laboratory for Reproductive Medicine of Guangdong Province, The First Affiliated Hospital of Sun Yat-Sen University, Guangzhou, China; Guangdong Provincial Clinical Research Center for Obstetrical and Gynecological Diseases, The First Affiliated Hospital of Sun Yat-Sen University, Guangzhou, China; Reproductive Medicine Center, The First Affiliated Hospital of Sun Yat-Sen University, Guangzhou, China; Key Laboratory for Reproductive Medicine of Guangdong Province, The First Affiliated Hospital of Sun Yat-Sen University, Guangzhou, China; Guangdong Provincial Clinical Research Center for Obstetrical and Gynecological Diseases, The First Affiliated Hospital of Sun Yat-Sen University, Guangzhou, China; Reproductive Medicine Center, The First Affiliated Hospital of Sun Yat-Sen University, Guangzhou, China; Key Laboratory for Reproductive Medicine of Guangdong Province, The First Affiliated Hospital of Sun Yat-Sen University, Guangzhou, China; Guangdong Provincial Clinical Research Center for Obstetrical and Gynecological Diseases, The First Affiliated Hospital of Sun Yat-Sen University, Guangzhou, China; Reproductive Medicine Center, The First Affiliated Hospital of Sun Yat-Sen University, Guangzhou, China; Key Laboratory for Reproductive Medicine of Guangdong Province, The First Affiliated Hospital of Sun Yat-Sen University, Guangzhou, China; Guangdong Provincial Clinical Research Center for Obstetrical and Gynecological Diseases, The First Affiliated Hospital of Sun Yat-Sen University, Guangzhou, China; Reproductive Medicine Center, The First Affiliated Hospital of Sun Yat-Sen University, Guangzhou, China; Key Laboratory for Reproductive Medicine of Guangdong Province, The First Affiliated Hospital of Sun Yat-Sen University, Guangzhou, China; Guangdong Provincial Clinical Research Center for Obstetrical and Gynecological Diseases, The First Affiliated Hospital of Sun Yat-Sen University, Guangzhou, China; Reproductive Medicine Center, The First Affiliated Hospital of Sun Yat-Sen University, Guangzhou, China; Key Laboratory for Reproductive Medicine of Guangdong Province, The First Affiliated Hospital of Sun Yat-Sen University, Guangzhou, China; Guangdong Provincial Clinical Research Center for Obstetrical and Gynecological Diseases, The First Affiliated Hospital of Sun Yat-Sen University, Guangzhou, China; Guangdong Provincial Clinical Research Center for Obstetrical and Gynecological Diseases, The First Affiliated Hospital of Sun Yat-Sen University, Guangzhou, China; Department of Obstetrics, The First Affiliated Hospital of Sun Yat-Sen University, Guangzhou, China; Department of Clinical Research, Yikon Genomics Co., Ltd, Suzhou, China; Department of Clinical Research, Yikon Genomics Co., Ltd, Suzhou, China; Department of Clinical Research, Yikon Genomics Co., Ltd, Suzhou, China; Department of Research and Development, Peking Jabrehoo Med-Tech Co., Ltd, Beijing, China; Department of Research and Development, Unimed Biotech (Shanghai) Co., Ltd, Shanghai, China; Department of Research and Development, Unimed Biotech (Shanghai) Co., Ltd, Shanghai, China; Department of Research and Development, Peking Jabrehoo Med-Tech Co., Ltd, Beijing, China; Reproductive Medicine Center, The First Affiliated Hospital of Sun Yat-Sen University, Guangzhou, China; Key Laboratory for Reproductive Medicine of Guangdong Province, The First Affiliated Hospital of Sun Yat-Sen University, Guangzhou, China; Guangdong Provincial Clinical Research Center for Obstetrical and Gynecological Diseases, The First Affiliated Hospital of Sun Yat-Sen University, Guangzhou, China; Reproductive Medicine Center, The First Affiliated Hospital of Sun Yat-Sen University, Guangzhou, China; Key Laboratory for Reproductive Medicine of Guangdong Province, The First Affiliated Hospital of Sun Yat-Sen University, Guangzhou, China; Guangdong Provincial Clinical Research Center for Obstetrical and Gynecological Diseases, The First Affiliated Hospital of Sun Yat-Sen University, Guangzhou, China

**Keywords:** PGT-A, mosaic, error origin, meiosis, embryo transfer, assisted reproduction

## Abstract

**STUDY QUESTION:**

What are the risk factors associated with meiotic errors in blastocysts with mosaic biopsy results?

**SUMMARY ANSWER:**

Meiotic errors were identified in 3.9% of blastocysts with mosaic biopsy results and were correlated with high-level mosaicism, maternal origin, and advanced maternal age.

**WHAT IS KNOWN ALREADY:**

Chromosomal mosaicism mainly arises from postzygotic mitotic errors, except for rare events from rescued meiotic errors. Preimplantation genetic testing for aneuploidy origin (PGT-AO) can distinguish meiotic errors from mitotic errors; however, the clinical value of PGT-AO remains to be explored.

**STUDY DESIGN, SIZE, DURATION:**

A retrospective cohort of 391 blastocysts with mosaic biopsy results from a university-based fertility centre in China between January 2020 and December 2024 was analysed by PGT-AO. Risk factors associated with meiotic errors in blastocysts with mosaic biopsy results were explored. Pregnancy outcomes following 96 mosaic embryo transfers (METs) of embryos with mitotic errors were compared with those following transfer of 288 matched euploid embryos. Additionally, 7 donated blastocysts were separated for single-cell DNA sequencing.

**PARTICIPANTS/MATERIALS, SETTING, METHODS:**

The parental origin and cell-division origin were analysed in 391 blastocysts classified as euploid–aneuploid mosaic from a cohort of 8932 blastocysts detected using the single-nucleotide polymorphism (SNP) array. In the prospective study, pregnancy outcomes following METs of embryos with mitotic errors were compared with those following transfer of matched euploid embryos at a 1:3 ratio using propensity score matching. The primary outcome was the live birth/ongoing pregnancy rate (LB/OPR) of METs involving mitotic errors. Multivariate logistic regression analysis was used to evaluate risk factors for pregnancy outcomes. Prenatal and placental samples were analysed by SNP array and/or FISH for genetic verification. Single-cell DNA sequencing of 314 cells separated from 7 donated blastocysts (4 with high-level mosaicism, 2 with meiotic aneuploidy, and 1 with low-level mosaicism) was conducted to assess actual mosaicism. The chromosomal constitution of the blastocysts was comprehensively evaluated at the single-cell level, and concordance with the initial PGT-A results was assessed.

**MAIN RESULTS AND THE ROLE OF CHANCE:**

A SNP-based mosaicism quantification platform was established and validated using mixtures of single cells of varying ploidy to mimic clinical mosaic samples. In the retrospective cohort, the error origin was successfully determined for 384 of the 391 blastocysts with euploid–aneuploid mosaicism. The overall meiotic error rate was only 3.9% (15/384). Meiotic error was identified in 9.6% of blastocysts with mosaicism from women of advanced maternal age, which was significantly greater than the 3.0% observed in blastocysts with mosaicism from young women (OR = 3.43, 95% CI 1.12–10.46; *P *= 0.039). In addition, meiotic error was identified in 7.2% (10/139) of blastocysts with high-level mosaicism but 2.0% (5/245) of blastocysts with low-level mosaicism (OR = 3.72, 95% CI 1.25–11.12; *P *= 0.012). Meiotic error was also significantly greater in blastocysts with maternal-origin mosaicism than in those with paternal-origin mosaicism (8.9% vs 0.4%, OR = 21.66; *P *< 0.001). Clinical outcomes were comparable between METs and euploid embryo transfers, with no significant difference in LB/OPR (47.9% vs 52.8%, *P *= 0.409). Single-cell sequencing of donated blastocysts with high-level mosaicisms of mitotic errors demonstrated highly variable mosaic levels, ranging from a reproducibility rate of 8.7–100% for initial mosaic abnormalities at the single-cell level.

**LARGE SCALE DATA:**

Due to the individual privacy of the patients, the data are not publicly available.

**LIMITATIONS, REASONS FOR CAUTION:**

Intrinsic technical noise cannot be completely distinguished from genuine mosaicism in the PGT-AO platform. The sample size of the MET cohort was limited, with few cases undergoing prenatal/postnatal genetic validation. In the single-cell study, the number of verified embryos was relatively small, and the threshold of copy number variation (CNV) detection was 10 Mb, which may have led to the underestimation of CNVs under 10 Mb.

**WIDER IMPLICATIONS OF THE FINDINGS:**

PGT-AO analysis demonstrated a low prevalence of meiotic errors in human blastocysts with mosaic biopsy results. PGT-AO is recommended for embryos from patients of advanced maternal age, embryos with high-level mosaicism, and embryos with maternal-origin mosaicism in PGT cycles.

**FUNDING:**

This study was supported by grants from the National Key Research and Development Program of China (No. 2023YFC2705503), National Natural Science Foundation of China (No. 82071716), Natural Science Foundation of Guangdong Province (No. 2025A1515010982), Key Clinical Technique of Guangzhou (No. 2023P-ZD19), and Medical Scientific Research Foundation of Guangdong Province (No. A2025203).

**DISCLOSURES:**

All authors declare no conflicts of interest.

WHAT DOES THIS MEAN FOR PATIENTS? Embryos created during IVF are sometimes found to have a mix of normal and abnormal cells after genetic testing. These are known as mosaic embryos. The mixed cell state can arise from errors that occur either before fertilization (in the oocyte or sperm) or after fertilization (as the embryo begins to divide). Transfer of embryos with errors before fertilization is doomed to end in IVF failure or miscarriage.

In our study, we used a detailed genetic test to work out when these errors occur. We found that errors before fertilization were uncommon, seen in just 3.9% of mosaic embryos. These errors were more likely to be seen in women aged 35 years or older, and in embryos with a large proportion of abnormal cells. Importantly, mosaic embryos with errors after fertilization had pregnancy outcomes similar to normal embryos after embryo transfer. However, we still strongly recommend prenatal diagnosis if a baby is conceived after mosaic embryo transfer. Our findings may help clinicians and patients make decisions when facing embryos with mosaic results.

## Introduction

Chromosomal mosaicism in a human embryo is when there is more than one population with distinct chromosomal constituents (Delhanty *et al.*, 1993; van Echten-Arends *et al.*, 2011). With advances in preimplantation genetic testing for aneuploidy (PGT-A), mosaic results based on intermediate copy number (CN) have been increasingly identified, varying from 2% to 40% at the blastocyst stage (Fragouli *et al.*, 2019; Leigh *et al.*, 2022). Mosaicism arises primarily from chromosome segregation errors during postzygotic mitosis (Popovic *et al.*, 2020). In embryos derived from normal gametes, the timing of mitotic errors and the cell lineages involved determine the proportion of abnormal cells (Taylor *et al.*, 2014; Waldvogel *et al.*, 2024). In rare cases, mosaicism occurs when a subset of aneuploid cells of meiotic aneuploid embryos undergoes trisomy or monosomy rescue to restore their euploidy (Popovic *et al.*, 2020), which may lead to severe consequences if these embryos are transferred into the uterus.

The recently proposed strategy of PGT for aneuploidy origin (PGT-AO) uses single-nucleotide polymorphism (SNP) haplotypes to determine the parental and mitotic/meiotic origins of chromosomal abnormalities (Zamani Esteki *et al.*, 2015; Verdyck *et al.*, 2022; Janssen *et al.*, 2024) and offers more comprehensive genome evaluation of the entire embryo. Among the principal PGT-A platforms, next-generation sequencing (NGS) allows the quantification of putative mosaicism via an assessment of intermediate CNs. However, conventional NGS is not capable of PGT-AO analysis because of its low sequencing depth. Alternative sequencing-based platforms, such as whole-genome sequencing (WGS) (Janssen *et al.*, 2024) and reduced representation sequencing (RRS) (De Witte *et al.*, 2022), enable analysis based on both CN and origin within a single PGT workflow. Nevertheless, WGS is costly for routine clinical use, whereas the resolution of RRS is limited because reads focus on scattered genomic regions. The SNP‑array platform enables genome-wide pedigree analysis using genotyping data from both parents and embryos. With more than 200 000 fixed SNP loci distributed across the genome, the SNP array platform is a practical and reliable approach for determining the origin of mosaicism (Zamani Esteki *et al.*, 2015; Tsuiko *et al.*, 2021). However, quantifying the level of mosaicism for trophectoderm (TE) biopsies on the SNP array platform is challenging, as no universal algorithm is currently available. Recently, one-third of 146 embryos initially diagnosed as mosaic by NGS were reported to originate from meiotic errors, as determined by SNP-based reanalysis (Popa *et al.*, 2025). In this study, mosaicism was defined as CN deviations between 30% and 80%, but some previous studies considered deviations above 70% to indicate aneuploidy (Jin *et al.*, 2026). Furthermore, the use of distinct detection platforms, variability in analytical algorithms across laboratories, and technical artefacts may complicate the interpretation of mosaicism. The real prevalence of meiotic error in PGT-A-identified mosaic results remains unclear.

The clinical significance and potential risks of blastocyst mosaicism identified by PGT-A remain controversial. Although retrospective studies on mosaic embryo transfers (METs) have reported reduced live birth rates (LBRs) and increased miscarriage rates compared with those following euploid transfers (Viotti *et al.*, 2021, 2023), prospective non-selection studies have shown comparable outcomes between low-level mosaic embryos (<50%) and euploid embryos (Capalbo *et al.*, 2021). Accordingly, ESHRE recommends selecting euploid and low-level mosaic embryos by evaluating both embryo morphology and PGT-A results (De Rycke *et al.*, 2022). However, rare cases of true foetal mosaicism or non-mosaic aneuploidy have been documented following the transfer of blastocysts with a mosaic result (Barad *et al.*, 2022; Schlade-Bartusiak *et al.*, 2022; Greco *et al.*, 2023). Furthermore, the risk of transferring embryos with high-level mosaicism remains unclear because the data are limited. To date, although several studies have employed an origin analysis to assess chromosomal abnormalities in biopsy specimens, the clinical value of PGT-AO in embryo selection and the prediction of pregnancy outcomes after METs remains to be established.

This study aimed to identify risk factors for meiotic errors in blastocysts with mosaic biopsy results and to assess clinical outcomes following MET of embryos with mitotic errors. We first optimized an SNP-based workflow integrating PGT-AO with CN analysis from a single biopsy. Using this method, we determined parental and meiotic/mitotic origins among 391 putative mosaic blastocysts. Pregnancy outcomes following 96 METs of embryos with confirmed mitotic errors were prospectively compared with those following 288 matched euploid transfers. Postnatal genetic validation was performed using cord blood and placental samples from nine MET pregnancies, and the products of conception from six miscarriages were assessed. Additionally, single-cell DNA sequencing of seven blastocysts was conducted to evaluate their chromosomal constitution. Together, our findings are expected to deepen our understanding of mosaic embryos and inform the clinical application of PGT-AO.

## Materials and methods

### Ethical approval

The study was approved by the Ethics Committee of the First Affiliated Hospital of Sun Yat-Sen University (No. [2020]110 and No. [2021]698). All the embryos used for this study were donated by patients after obtaining written informed consent. Clinical data have been deidentified.

### Study setting and participants

**Figure 1.**
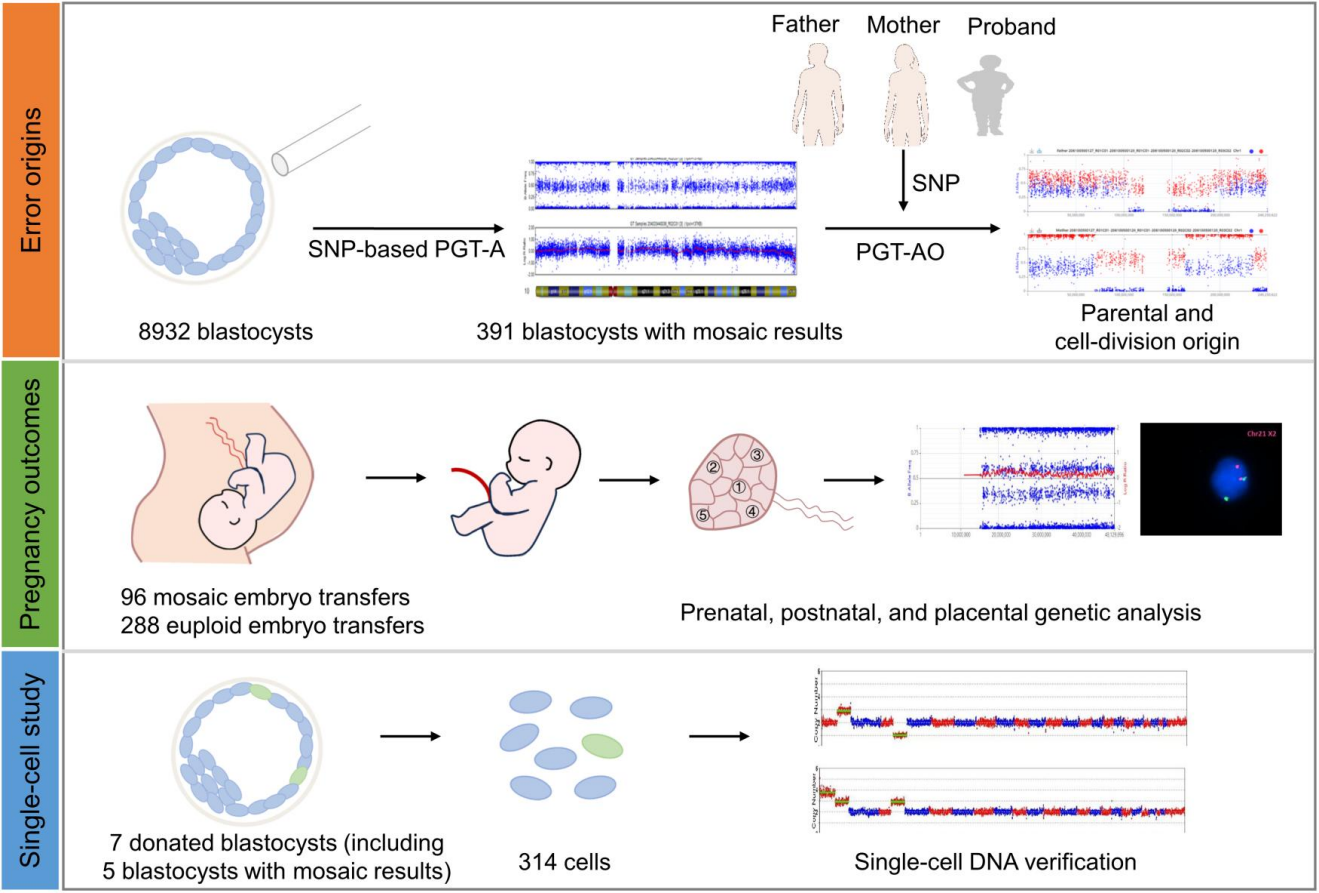
**Workflow of the study on preimplantation genetic testing for aneuploidy origin in human blastocysts with mosaic results.** The parental and cell-division origins of 391 blastocysts identified as euploid–aneuploid mosaics among 8932 blastocysts were analysed using SNP arrays. Second, the pregnancy outcomes of 96 mosaic embryo transfers with mitotic errors were compared with those of 288 matched euploid embryo transfers, with comprehensive genetic assessment of prenatal, postnatal, and placental samples. Finally, single‑cell DNA sequencing on 314 cells isolated from 7 donated blastocysts was performed to validate the actual extent of mosaicism. PGT-A, preimplantation genetic testing for aneuploidy; PGT-AO, preimplantation genetic testing for aneuploidy origin; SNP, single-nucleotide polymorphism.

Embryo data were obtained from the Reproductive Medicine Center, the First Affiliated Hospital of Sun Yat-Sen University. The retrospective cohort included 8932 blastocysts from 1626 PGT cycles that underwent comprehensive chromosomal screening with an SNP array platform from January 2020 to December 2024. Among these, 391 blastocysts from 339 cycles of 326 couples were identified as having putative mosaicism and were analysed by PGT-AO to determine their parental and cell-division origins. Pregnancy outcomes and prenatal/postnatal genetic analyses were carried out for 96 MET cycles determined to be of mitotic origin. The control group was obtained from 5249 transfer cycles of embryos classified as euploid conducted during the same period. Owing to the technical limitations of the SNP array platform at a single-cell resolution, blinded NGS was performed to detect chromosomal constitution in individual cells isolated from seven donated blastocysts (four with high-level mosaicism, two aneuploid, and one with low-level mosaicism) from PGT for monogenic disorder (PGT-M) or structural rearrangement (PGT-SR) cycles. Single-cell results were analysed to assess their concordance with initial TE biopsy results (Fig. 1, Supplementary Fig. S1).

### Clinical TE biopsy, embryo vitrification, and warming

Controlled ovarian stimulation, oocyte retrieval, ICSI, embryo culture, and TE biopsy were performed following routine clinical procedures (Liu *et al.*, 2020). Blastocyst quality was classified on the basis of morphology using Gardner’s grading system (Gardner and Schoolcraft, 1999) with the following classifications: good (≥3 AA, AB, and BA), average (≥3 BB, AC, and CA), and poor (≥3 BC and CB). All the enrolled embryos originated from fresh cycles and underwent a single biopsy. After biopsy, the embryos were immediately vitrified using the Cryotop method (Kuwayama, 2007). Following warming, the embryos were transferred into the uterus. For single-cell analysis, the embryos were cultured for 12–24 hours prior to single-cell separation.

### SNP array platform and CN analysis

Whole-genome amplification (WGA) of clinical TE biopsies was conducted using the REPLI-g Single-Cell WGA Kit (Qiagen, Hilden, Germany) based on multiple displacement amplification (MDA) methods. Peripheral blood from parents and siblings was collected for pedigree analysis. When a proband was unavailable, sibling blastocysts from the same cycle served as references. Genomic DNA (gDNA) extraction was performed using the TGuide Blood Genomic DNA Kit (TIANGEN, Beijing, China). SNP genotyping of WGA products and gDNA was carried out using the Human CytoSNP-12/Karyomap-12 Bead Chip with ∼300 000 SNP loci (Illumina, San Diego, CA, USA). The hybridized chips were scanned with the Illumina iScan reader, and the data were analysed with GenomeStudio software (Illumina, San Diego, CA, USA).

Chromosome ploidy was first assessed on the basis of BAF and LogR. Mosaicism was defined by a scattered BAF distribution for heterozygous SNPs from 0.5 and altered LogR (|LogR|<0.2), indicating a putative mosaic embryo (Rodriguez-Santiago *et al.*, 2010). Second, to achieve precise mosaicism quantification of TE biopsies, we developed a quantification method combining SNP data conversion with NGS-based algorithms. Briefly, the SNP data were converted to variant call format using the IAAP Genotyping Command Line Interface and gtc-to-vcf conversion (Illumina). CN analysis was then performed on a conventional NGS platform (Peking Jabrehoo, Beijing, China). SNP loci were divided into consecutive bins (600 kb) and normalized for GC content, mappability, and baseline. The hidden Markov model was used for joint segmentation. CN values were derived from the median normalized value of all windows. Copy number variants (CNVs) larger than 4 Mb were reported. Intermediate CN was classified by CN values between 1.3–1.7 or 2.3–2.7, indicating an abnormal cell ratio of 30–70%. Mosaicism levels were calculated as |CN − 2|×100%, with levels ≥50% classified as high-level mosaicism. Together, a mosaic result on TE biopsy was defined by the presence of scattered BAF distribution in conjunction with intermediate CN signals.

### Validation of mosaicism level

Validation of mosaicism quantification was performed through three approaches (Supplementary Fig. S2A). First, we utilized single-cell mixture models with lymphocytes from three mother–son pairs. All included patients had a normal karyotype. Lymphocytes were isolated via Ficoll separation (Solarbio, Beijing, China), washed, and diluted into single-cell suspensions. To mimic a TE biopsy, 10-cell samples comprising a series of lymphocytes from the mother and son with varying mosaic proportions (0%, 30%, 50%, 70%, 100%), i.e. 10 cells from the mother and 0 cells from the son representing 0% mosaicism in chromosome Y and 5 cells from the mother and 5 cells from the son representing 50% mosaicism in chromosome Y, were constructed via micromanipulation. These samples were subsequently subjected to WGA and SNP array analysis, and the mosaic levels of chromosomes X and Y calculated by our quantitative method were compared with the actual mixing ratios. The experiments were repeated three times.

Second, we estimated the detection accuracy in different aneuploid cell lines with predefined mixing ratios (Supplementary Fig. S2D). Each experiment contained six cells per tube constructed via micromanipulation. Aneuploid cell lines were obtained from the Human Genetic Cell Repository of the National Institute of General Medical Sciences (Coriell Institute, Camden, NJ, USA). Whole-chromosome aneuploidy was examined in a cell line with trisomy 21 (GM02767). Segmental chromosome abnormalities were detected in cell lines with 7q deletion (GM01059, 25.27 Mb), 10q deletion (GM05299, 14.14 Mb), and 10p deletion (GM21877, 11.9 Mb). We established six-cell models via micromanipulation with predefined aneuploid cell ratios (2:4, 3:3, and 4:2). The performance of mosaicism quantification for targeted chromosomes was compared with the known mixing ratios across 10 experimental replicates. Additionally, reserved WGA products from TE biopsies of 80 blastocysts, initially diagnosed as mosaic by the routine NGS platform, were reanalysed on our SNP-based quantitative platform. The concordance of mosaicism calling between the two methods was assessed by the intraclass correlation coefficient (ICC) and Bland–Altman plots (Supplementary Fig. S2E).

### PGT-AO and validation of the PGT-AO results

The parental genotypes were phased on the basis of the proband genotypes to determine the genome-wide haplotypes. Paternal and maternal informative SNPs were identified, and the BAF distribution of parental informative SNPs was utilized to analyse the parental and cell-division origins of the embryos, as described previously (Zamani Esteki *et al.*, 2015). Briefly, a parental informative SNP locus was identified when one parent was homozygous and the other parent was heterozygous. On the basis of phased parental genotype combinations, paternal and maternal informative SNPs were subcategorized as P1/P2 and M1/M2. Combined with CN, the distance of BAF values between P1 and P2 (or ‘M1 and M2’) determined parental and mitotic/meiotic origins according to unique haplotype patterns of different chromosomal abnormalities. With respect to chromosomal gains, both parental homologue (BPH) patterns inherited from one parent indicated meiotic errors, whereas single-parental homologue (SPH) signatures suggested mitotic errors or meiotic errors without recombination. Errors during meiosis I and meiosis II were distinguished on the basis of BPH and centromere positioning (Supplementary Fig. S3). With respect to chromosomal losses, BAF values near 0 or 1 at informative SNP loci in the affected parent indicated complete loss of heterozygosity, suggesting a meiotic error. In contrast, a mitotic origin was characterized by the presence of heterozygous pattern of informative SNP loci from both parents, with BAF values distributed between 0 and 0.5 or between 0.5 and 1 (Rana *et al.*, 2023; De Witte *et al.*, 2024). To minimize amplification noise, embryos (n = 12) involving >3 chromosomes with mosaicism or embryos (n = 7) with a call rate lower than 0.90 were excluded from the analysis.

To validate the ability of the PGT-AO platform to determine the origin of chromosomal abnormalities, we reanalysed 15 TE samples from three reciprocal translocation carrier couples as positive controls. These samples had previously been analysed using Karyomapping and diagnosed with unbalanced segregation of the translocated chromosomes using BlueFuse software (Illumina). Furthermore, to compare the error origin detection performance between the platforms, 69 trisomies initially detected by BlueFuse were reanalysed using our PGT-AO platform.

### Prospective study of METs

In this prospective study, blastocysts with mosaicism classified by PGT-A underwent frozen-thawed embryo transfer (FET) after comprehensive genetic counselling from January 2020 to May 2025. The study groups included 96 cycles that transferred embryos identified as mosaic of mitotic origin by PGT-AO. The control group was obtained from 5249 transfer cycles of euploid embryos conducted during the same period. To minimize group size differences, propensity score matching (PSM) was used to match the control group at a 1:3 ratio and a calliper width of 0.2 using the nearest neighbour matching method. Univariate analysis was used to compare the baseline characteristics among groups. Demographic baseline data, cycle characteristics, and covariates associated with transfer outcomes, including maternal age, BMI, basal FSH, gravidity, PGT indication, ovarian stimulation protocol, the number of retrieved oocytes and biopsied blastocysts, PGT platform, endometrial preparation, prior transfer cycles, and blastocyst quality and developmental speed, were included in the PSM models. FET and luteal support protocols were performed according to routine clinical procedures (Liu *et al.*, 2020). Prenatal diagnosis results and pregnancy outcomes were obtained from medical records in our hospital or by telephone follow-up. Pregnancy outcomes were compared among METs of embryos with mitotic errors and euploid embryo transfers (EETs), and subgroup analyses were conducted on the basis of mosaicism of parental origin and maternal age. Multivariate logistic regression analyses were employed to adjust for potential confounders. Gestational week was calculated according to the day of blastocyst transfer. The primary outcome was the live birth/ongoing pregnancy rate (LB/OPR). Clinical pregnancy was confirmed by the presence of a gestational sac on transvaginal ultrasound at 7 weeks of gestation. Miscarriage was defined as pregnancy loss during a clinical pregnancy before 20 weeks of gestation. Umbilical cord blood and multisite placental samples were collected from nine mosaic cases for postnatal validation by chromosomal microarray analysis (CMA) and FISH, with written informed consent from parents. FISH was performed according to the manufacturer’s instructions (CytoCell, Cambridge, UK). Products of conception in miscarriage cases were analysed by CMA.

### Single-cell DNA sequencing of blastocysts

Single-cell verification of seven donated blastocysts (four with high-level mosaicism, two with meiotic aneuploidy, and one with low-level mosaicism) was performed. Inner cell mass (ICM) and TE cells were mechanically separated using microsurgical techniques. Briefly, the zona pellucida was removed by hyaluronidase treatment and then immobilized with a holding pipette. A biopsy pipette was inserted into the blastocoel, after which the ICM cells were removed with laser assistance. The ICM cells and the remaining clumps of TE cells were dissociated into single-cell suspensions via trypsin treatment at 37°C for 15 min. Single-cell samples were separately transferred into a tube containing 5 μl of lysis buffer and stored at −80°C until WGA processing.

For single-cell DNA sequencing, WGA and library construction were conducted using the ChromInst Library Preparation Kit (Yikon Genomics, Suzhou, China) (Gao *et al.*, 2021). Amplified products were subjected to sequencing with single-end reads (55 bp) on the NextSeq 550 platform (Illumina). At least 1 million raw reads were obtained per cell, yielding ∼2% genome coverage. Reads were counted across the whole genome in 1-Mb bins, normalized by GC content and against a reference dataset, using the circular binary segmentation algorithm. Given amplification bias from single-cell processing, only CNVs larger than 10 Mb were reported, and CN values ranging from 1.3 to 1.7 or 2.3 to 2.7 were classified as a putative intermediate CN signal. Single cells from the trisomy 21 cell lines were used as positive controls. We selected one non-mosaic blastocyst with four segmental abnormalities from a male carrier with two reciprocal translocations (Embryo 1) to verify the resolution of the single-cell detection platform. Our results revealed a 100% concordance rate across all single cells, indicating the high sensitivity and specificity of our single-cell platform. A total of 364 single cells were isolated. After quality control, 314 cells (86.3%), comprising 118 ICM cells and 196 TE cells, were successfully sequenced and analysed. The chromosomal constitution of the blastocysts was evaluated at single-cell resolution, and its concordance with the initial PGT-A results was assessed.

### Statistical analyses

SPSS 26.0 software (IBM, Amonk, NY, USA) was used for statistical analyses. Continuous variables are presented as the mean and SD or median with interquartile range and were compared using Student’s *t*-test or the Mann–Whitney *U*-test, as appropriate. Categorical variables are summarized as frequencies (percentages) and were analysed with the binomial, chi-square, or Fisher’s exact test. Data processing and PSM analysis were performed using R version 4.3.3 and Zstats v1.0 (www.zstats.net, Hangzhou, China). Factors associated with pregnancy outcomes following METs were identified by multivariable logistic regression. A *P*-value <0.05 was considered to indicate statistical significance.

## Results

### Validation of the SNP-based method for quantifying mosaicism level and distinguishing the error origin

First, SNP-based mosaicism quantification was validated in both sex chromosomes and autosomes. A mosaic model comprising mixtures of lymphocytes from mother and son at different ratios (10-cell samples) was used to validate sex chromosomes. The results revealed less than 10% deviation in chromosome X detection from the actual mixing ratios (Supplementary Fig. S2B and C). Second, mosaic models with mixed aneuploid cell lines of autosomes were used for validation. Targeted chromosomes were accurately quantified, with a median deviation of 3% (range: −7% to 11.7%) between the detected and actual mixing ratios (Supplementary Fig. S2D). Additionally, WGA products from 80 mosaic blastocysts were analysed by both the SNP array and NGS platforms and demonstrated high concordance (ICC = 0.930; 95% CI 0.893–0.955). Bland–Altman analysis revealed minimal bias (mean difference = −0.64%, 95% CI −1.97% to 0.69%), with limits of agreement ranging from −12.3% to 11.1% (Supplementary Fig. S2E). These results confirmed the reliability of the SNP-based quantification approach.

The accuracy of PGT-AO for origin determination was assessed in 15 unbalanced embryos from reciprocal translocation carriers, confirming the presence of a meiotic I error of carrier parental origin. A comparative analysis between the PGT-AO platform and BlueFuse software revealed the consistent detection of all meiotic-origin trisomies across both platforms. Notably, PGT-AO successfully determined the parental origin of 15 mitotic-origin trisomies that remained undetermined by BlueFuse (Supplementary Table S1, Supplementary Fig. S4). PGT-AO thereby addressed the inherent limitation of BlueFuse in visualizing SPH patterns within chromosomal gains in the haploblock chart (Kubicek *et al.*, 2019).

### Cohort derivation for the PGT-AO analysis

Among 8932 blastocysts derived from 1626 oocyte retrieval cycles that underwent SNP-based PGT, 500 (5.6%) exhibited a scattered BAF distribution. Subsequent CN quantification using a threshold of 30–70% classified 410 (4.6%) as euploid-aneuploid mosaic, 50 as aneuploid, and 40 as euploid. After 19 blastocysts with a low call rate or complex mosaicism were excluded, 391 blastocysts with mosaic results underwent PGT-AO. Of these, 384 (98.2%) blastocysts from 333 cycles clearly had defined parental and cell-division origins, whereas the remaining 7 (1.8%) blastocysts yielded ambiguous results and were excluded from further analysis (Supplementary Fig. S1). The mean maternal age at oocyte retrieval cycles was 30.7 ± 3.4 years, and the mean paternal age was 35.6 ± 4.5 years.

### Parental and cell-division origins of mosaicisms in blastocysts

**Figure 2.**
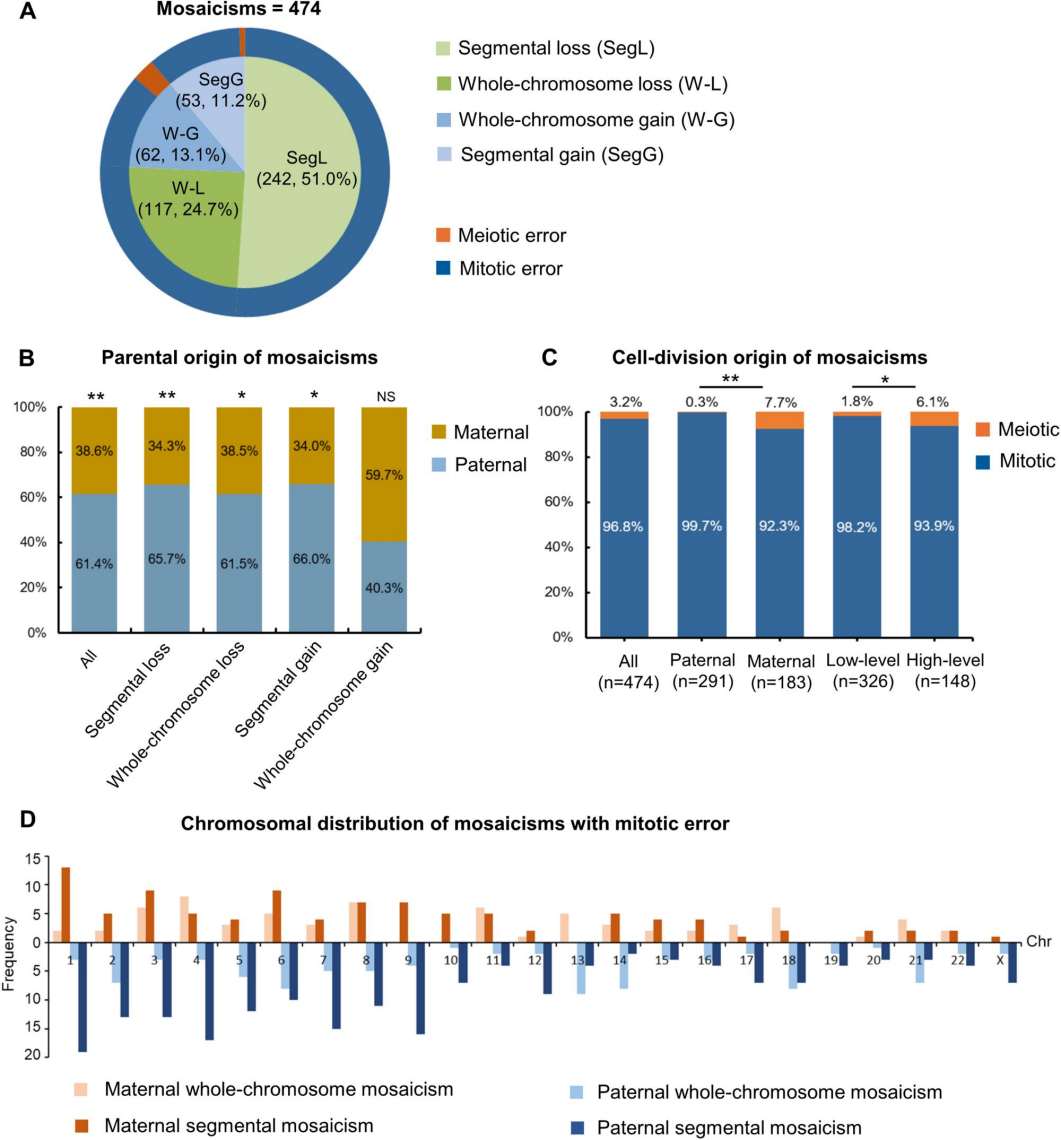
**Parental and cell-division origins of mosaicisms.** (**A**) Overview of the 474 individual mosaic events in 384 blastocysts detected by preimplantation genetic testing for aneuploidy origin. (**B**) Parental origin distribution of 474 mosaicisms and different subcategories of mosaicisms. (**C**) Cell-division origins of 474 mosaicisms, stratified by parental origin and mosaicism level. Mosaicism levels ≥50% were classified as high-level mosaicism. (**D**) Chromosome distribution of mosaicisms with mitotic origin (n = 459). *P*-values were calculated by binomial test (B), chi-square, and Fisher’s exact test (C). A *P*-value of <0.05 was considered statistically significant. *: *P*-value <0.05; **: *P*-value <0.01; NS, not significant. Chr, chromosome.

Among the 384 blastocysts with mosaic results, 59 (15.4%) blastocysts displayed more than one chromosomal abnormality, resulting in a total of 474 individual mosaicisms (Fig. 2A). Mosaicisms of paternal origin were more prevalent than those of maternal origin (61.4% vs 38.6%, *P *< 0.001). This paternal predominance was particularly pronounced in segmental mosaicisms, with paternal origin accounting for 65.8% of the mosaicisms and maternal origin accounting for 34.2% of mosaicisms (*P *< 0.001) (Fig. 2B). Chromosomal losses predominated over chromosomal gains in paternal segmental mosaicisms (82.0% vs 18.0%, *P *< 0.001). This paternal bias was not observed for whole-chromosome mosaicisms (Fig. 2B).

Analysis of cell-division origins of mosaicisms revealed that all mosaic chromosomal losses (75.7%, 359/474) displayed heterozygous SNP patterns, indicating their mitotic origins (De Witte *et al.*, 2024). Among 115 mosaic chromosomal gains, 13.0% (15/115) exhibited BPH patterns, indicating meiotic errors, whereas 87.0% (100/115) showed SPH patterns, suggesting either mitotic errors or meiotic errors without recombination (Fig. 2A). Given the rarity of nonrecombinant meiosis (Halldorsson *et al.*, 2019), meiotic errors accounted for only 3.2% (15/474) of mosaic abnormalities in this cohort, while the majority (96.8%, mosaic losses and SPH gains) originated from mitotic errors. The meiotic error rate (6.1%, 9/148) was significantly greater for high-level mosaicisms than for low-level mosaicisms (1.8%, 6/326) (OR = 3.45, 95% CI 1.21–9.89; *P *= 0.031) and significantly greater in maternal-origin mosaicisms than in paternal-origin mosaicisms (7.7% vs 0.3%, OR = 24.02, *P *< 0.001) (Fig. 2C). When mosaic chromosomal gains alone were considered, meiotic errors accounted for 25.5% of maternal-origin mosaic gains but only 1.7% of paternal-origin mosaic gains (*P *< 0.001). Chromosome 22 was the most frequently involved chromosome with meiotic-origin mosaicisms (Supplementary Table S2). An uneven distribution of mitotic-origin mosaicisms was observed across the genome, with paternal segmental mosaicisms frequently affecting large chromosomes, accounting for 64.9% of those on chromosomes 1–9 (Fig. 2D).

Next, cell-division origins of mosaicisms were analysed at the embryo level. Among the 384 blastocysts with mosaicism identified on biopsy, the overall rate of meiotic error was 3.9% (15/384, Table 1). Blastocysts with mosaicism of maternal origin (8.9%, 12/135) and blastocysts containing multiple mosaicisms of both maternal and paternal origin (7.7%, 2/26) were associated with a significantly higher incidence of meiotic errors than those of paternal origin (0.4%, 1/223, *P *< 0.001). Additionally, compared with that in blastocysts with low-level mosaicism, the rate of meiotic error was 3.7-fold greater in blastocysts with high-level mosaicism (7.2% vs 2.0%, odds ratio (OR) = 3.72; 95% CI 1.25–11.12; *P *= 0.012). Meiotic error rates in embryos showing mosaicism significantly increased with increasing maternal age: the meiotic error rate was 3.0% with maternal age of <35 years, 8.3% with maternal age of 35–37 years, and 12.5% with maternal age of over 38 years. In women of advanced maternal age (≥ 35 years), 9.6% of blastocysts with mosaicisms were of meiotic origin, a value that was significantly higher than that observed in young women (3.0%, OR = 3.43; 95% CI 1.12–10.46; *P *= 0.039).

**Table 1.** Incidence of meiotic error in human blastocysts identified as mosaic.

Characteristics	Embryos (n)	Meiotic error rate % (n)	OR	95% CI	*P*-value
Blastocysts with mosaicism	384	3.9 (15/384)	–	–	–
Parental origin	–	–	–	–	<0.001
Paternal origin	223	0.4 (1/223)	Reference	–	–
Maternal origin	135	8.9 (12/135)	21.66	2.78–168.55	<0.001
Both origins ^a^	26	7.7 (2/26)	18.50	1.62–211.64	0.030
Mosaicism level ^b^	–	–	–	–	0.012
Low-level	245	2.0 (5/245)	Reference	–	–
High-level	139	7.2 (10/139)	3.72	1.25–11.12	0.012
Maternal age ^c^	–	–	–	–	0.039
< 35 years	332	3.0 (10/332)	Reference	–	–
≥ 35 years	52	9.6 (5/52)	3.43	1.12–10.46	0.039

a Both paternal and maternal origin mosaicisms were identified in these blastocysts.

b Mosaicism levels ranging from 30% to 50% were classified as low-level mosaicism, and mosaicism levels ≥50% were classified as high-level mosaicism.

c Maternal age was tested at the oocyte retrieval cycle.

Statistical significances were analysed by chi-square test or Fisher exact test. The *P*-value <0.05 was considered statistically significant.

OR, odds ratio.

### Pregnancy outcomes of METs

During the study period, 96 embryos with confirmed mitotic origin and 5249 embryos classified as euploid were transferred. Before PSM, the groups differed in terms of PGT indication, biopsied embryo count, blastocyst quality, and developmental speed (Supplementary Table S3). METs of embryos with mitotic errors had an increased miscarriage rate compared with that of all EET cycles (19.7% vs 9.9%, *P *= 0.012). Following PSM, the MET group with mitotic origin and the matched euploid control group (n = 288) were well balanced across all baseline characteristics. No significant differences were observed in the clinical pregnancy rate (63.5% vs 61.1%, *P *= 0.671), miscarriage rate (19.7% vs 12.5%, *P *= 0.169), or LB/OPR (47.9% vs 52.8%, *P *= 0.409) between the MET group with mitotic origin and the control group (Table 2). On multivariable regression analysis, poor blastocyst quality independently predicted a lower LB/OPR, whereas both poor blastocyst quality and delayed development (Day 7 blastocysts) were independent risk factors for a reduced clinical pregnancy rate (Supplementary Table S4). Specifically, poor-quality mosaic blastocysts (grade C TE or ICM cells) presented lower clinical pregnancy rates than good-quality counterparts did, and grade C TE cells were associated with a lower LB/OPR than grade A TE cells were (*P *= 0.023).

**Table 2.** Pregnancy outcomes of mosaic embryo transfers with confirmed mitotic origin.

	METs with mitotic origin ^a^	Euploid embryo transfer before PSM ^b^	Euploid embryo transfer after PSM ^c^	*P*-value (a vs b)	*P*-value (a vs c)
All transfers, n	96	5249	288	–	–
Biochemical pregnancy, n (%)	66 (68.8)	3864 (73.6)	203 (70.5)	0.284	0.748
Clinical pregnancy, n (%)	61 (63.5)	3406 (64.9)	176 (61.1)	0.784	0.671
Miscarriage, n (%)	12 (19.7)	336 (9.9)	22 (12.5)	**0.012**	0.169
LB/OP, n (%)	46 (47.9)	3016 (57.5)	152 (52.8)	0.061	0.409

a Mosaic embryo transfers of embryos with mitotic errors.

b Euploid embryo transfers before PSM.

c Euploid embryo transfers after PSM.

Statistical significances were analysed by chi-square test. Bold values indicate statistical significance (*P*-value < 0.05).

LB/OP, Live birth/ongoing pregnancy; MET, mosaic embryo transfers; PSM, propensity score matching.

Among the 46 live birth/ongoing pregnancies from METs of embryos with mitotic errors, 31 foetuses underwent CMA and were confirmed to have a normal karyotype. Umbilical blood from neonates and multisite placental tissues were obtained for postnatal validation in eight live births. The SNP array confirmed euploidy in all postnatal samples. FISH analysis revealed low rates of abnormal signals in chromosomes corresponding to the mosaic findings on TE biopsy, ranging from 0% to 9% in umbilical blood and 0.8–6.8% in placental cells. These frequencies were within the expected range of technical variability compared with the data from euploid controls (Table 3). Notably, a case of foetal trisomy 21 was detected by amniocentesis after the transfer of a blastocyst initially reported as mosaic monosomy 21 of mitotic origin (mosaicism level 35%). The presence of trisomy 21 was confirmed in umbilical cord blood cells after termination. However, SNP array analysis of placental tissues revealed mosaic trisomy 21 varying from 31% to 100% across five sampling sites. Subsequent FISH analysis revealed a predominance of trisomy 21 cells in placental samples, accompanied by diploid and sporadic monosomy 21 cells. Chromosomal abnormalities in all the samples were identified as having a paternal mitotic error (Supplementary Fig. S5). Among the 12 miscarriages following METs of embryos with mitotic errors, products of conception were available for genetic analysis in six cases. Two of these six cases exhibited an abnormal karyotype, demonstrating either uniform segmental aneuploidy or high-level mosaicism corresponding to the chromosomal abnormalities initially detected on TE biopsy (Table 3). The origin analysis confirmed the mitotic origin of these abnormalities in the products of conception.

**Table 3.** Genetic validations of embryos in conceived pregnancies.

Group	Pregnancies (n)	Genetic validations performed, n (%)	Prenatal diagnosis results (CMA)	POC results (CMA)	Postnatal results (CMA/FISH ^a^)	Multisite placental results (CMA/FISH ^a^)
MET of mitotic origin (n = 96)						
Live birth/ongoing pregnancy	46	31 (67.4%)	100% Normal	–	CMA: 100% normal FISH: abnormal signals in 0–9% of cells ^b^	CMA: 100% normal FISH: abnormal signals in 0.8–6.8% cells ^b^
Miscarriage	12	6 (50.0%)	–	66.7% Normal ^c^	–	–
Termination of pregnancy	1	1	Trisomy 21	–	CMA: Trisomy 21 FISH: Trisomy 21	CMA: Mosaic trisomy 21 FISH: Mosaic trisomy 21
Euploid embryo transfers (n = 288)						
Live birth/ongoing pregnancy	152	101 (66.4%)	100% Normal	–	–	–
Miscarriage	22	8 (36.4%)	–	87.5% Normal ^d^	–	–
Termination of pregnancy	2	1	Normal	–	–	–

a Postnatal and multisite placental validation was performed for eight live births after mosaic embryo transfers using CMA and FISH analysis.

b FISH was used to analyse the signals in PGT-A-identified mosaicism in umbilical cord blood and placental cells. In each FISH experiment, FISH probes targeting karyotypically normal chromosomes were used as controls, and signal deviations were observed in 0–10% of cells, which were considered technical artifacts.

c Two miscarriages after mosaic embryo transfers showed either uniform segmental aneuploidy or high-level mosaicism corresponding to the chromosomal mosaicism initially detected in TE biopsies.

d POC from one miscarriage following euploid embryo transfer was detected with segmental chromosomal gains and segmental mosaicism.

CMA, chromosomal microarray analysis; MET, mosaic embryo transfer; POC, products of conception.

### Single-cell DNA sequencing of human blastocysts

To explain the unexpected trisomy 21 pregnancy following the transfer of a blastocyst with low-level mosaicism and two miscarriages with an abnormal karyotype, single-cell DNA sequencing was performed to investigate the chromosomal constitution of the embryos and its correlation with their cell-division origins. Four blastocysts with high-level mosaicism and three blastocysts with no or low-level mosaicism on biopsy were analysed (Table 4). In three blastocysts with meiotic errors identified by PGT-AO, initial chromosomal abnormalities on TE biopsy results were confirmed in all single cells. Specifically, in a blastocyst with meiotic error mosaicism on biopsy, 90% (27/30) of ICM cells and all TE cells showed full concordance, whereas 10% (3/30) of ICM cells exhibited partial concordance, with variable segmental lengths on the same chromosome (Table 4).

**Table 4.** Comparison of the initial PGT results with single-cell results of donated blastocysts.

Embryo	PGT-AO results					Single-cell results			
	Karyotype	Chromosome/region	Mosaicism level	Parental origin	Cell-division origin	Number of single cells	Cells with initial aneuploidy, n (%)	Cells with initial mosaicism, n (%)	Cells with *de novo* abnormality, n (%)
Embryo 1	46, XY, del 3q25.1, dup 6p12.3→qter, dup 7p21.3, del 11p13→qter	3q25.1 6p12.3→qter 7p21.3 11p13→qter	– – – –	Pat Pat Pat Pat	Meiotic I Meiotic I Meiotic I Meiotic I	Total: 22 ICM: 8 TE: 14	22 (100) 8 (100) 14 (100)	–	3 (13.6) 2 (25.0) 1 (7.1)
Embryo 2	46, XX, +22	22	–	Mat	Meiotic I	Total: 94 ICM: 30 TE: 64	94 (100) 30 (100) 64 (100)	–	13 (13.8) 4 (13.3) 9 (14.1)
Embryo 3	46, XX, mos +1	1	62%	Pat	Meiotic II	Total: 57 ICM: 30 TE: 27	–	57 (100) 30 (100) 27 (100)	7 (12.3) 7 (23.3) 0 (0)
Embryo 4	47, XX, +3, mos +11	3 11	– 60%	Pat Mat	Mitotic Mitotic	Total: 53 ICM: 15 TE: 38	53 (100) 15 (100) 38 (100)	53 (100) 15 (100) 38 (100)	12 (22.6) 4 (26.7) 8 (21.1)
Embryo 5	46, XY, mos −18	18	67%	Pat	Mitotic	Total: 33 ICM: 11 TE: 22	–	14 (42.2) 4 (36.4) 10 (45.4)	11 (33.3) 3 (27.3) 8 (36.4)
Embryo 6	46, XX, dup 5q21.3, del 6p22.1, mos −9q	5q21.3 6p22.1 9q	– – 69%	Pat Pat Pat	Meiotic I Meiotic I Mitotic	Total: 23 ICM: 8 TE: 15	23 (100) 8 (100) 15 (100)	2 (8.7) 1 (12.5) 1 (6.7)	7 (30.4) 2 (25.0) 5 (33.3)
Embryo 7	SNP array: 47, XX, mos −12, mos −17, +21 NGS: 47, XX, mos −13, +21	12 *^a^* 13 17 ^*a*^ 21	14% 41% 16% –	Mat Un Mat Mat	Mitotic Mitotic Mitotic Meiotic I	Total: 32 ICM: 16 TE: 16	32 (100) 16 (100) 16 (100)	1 (3.1) 1 (6.3) 0 (0)	5 (15.6) 3 (18.8) 2 (12.5)

a BAFs of heterozygous SNP loci in chromosome 12 and 17 were deviated from 0.5, but the calculated mosaicism level was lower than 30%. These chromosomes were also considered a putative mosaicism for single-cell analysis.

del, deletion; dup, duplication; ICM, inner cell mass; Mat, maternal; mos, mosaic; NGS, next-generation sequencing; Pat, paternal; PGT, preimplantation genetic testing; PGT-AO, preimplantation genetic testing for aneuploidy origin; SNP, single-nucleotide polymorphism; TE, trophectoderm; Un, undefined.

In blastocysts diagnosed with high-level mosaicisms of mitotic origin, the reproducibility of mosaic abnormalities varied greatly among single cells (Supplementary Table S5). Initial mosaic abnormalities were detected in 8.7–100% of single cells per embryo, ranging from 12.5% to 100% in ICM cells and from 6.7% to 100% in TE cells (Table 4). Notably, Embryo 4 exhibited trisomy 3 and high-level mosaic trisomy 11 originating from mitotic errors on PGT diagnosis, yet all single cells were confirmed to have uniform aneuploidies for both chromosomes (Supplementary Fig. S6). In Embryo 7 with low-level mosaicism, the reproducibility rate was only 3.1%.

In addition to PGT-A-reported abnormalities, *de novo* abnormalities were detected in all analysed blastocysts in single-cell analysis, affecting an average of 20.2% (range 12.3–33.3%) of the cells per embryo. This finding aligned with previous single-cell sequencing studies describing such events in most or even all of the embryos (Chavli *et al.*, 2024; Zhai *et al.*, 2024). No significant differences were observed between ICM cells (22.7%) and TE cells (17.8%, *P *= 0.50). *De novo* events occurred sporadically on most chromosomes except for chromosome 11. Reciprocal chromosomal gains and losses in identical regions occurred in 57.1% (4/7) of the embryos, involving 5.3–13.2% of the cells per embryo (Supplementary Fig. S7).

## Discussion

The clinical importance of mosaicism in PGT-A biopsies remains to be clarified (Practice Committee and Genetic Counseling Professional Group of the American Society for Reproductive Medicine, 2020). Our study demonstrated that only 3.9% of blastocysts with mosaic biopsy results contained meiotic errors and were correlated with advanced maternal age, high-level mosaicism, and maternal origin. In addition, compared with euploid blastocyst transfers, METs of embryos with mitotic errors had comparable pregnancy outcomes.

Given that aneuploidy of meiotic origin is a major cause of implantation failure and pregnancy loss (Nagaoka *et al.*, 2012), the transfer of embryos with mosaic PGT results but true aneuploidies should be avoided in the clinic. Our results demonstrated that meiotic error contributed to only 3.9% of human blastocysts with mosaic biopsy results, indicating that most mosaicisms identified by SNP-based PGT-A originate from postzygotic mitotic errors. This may account for the observation that METs did not substantially compromise clinical pregnancy or live birth outcomes (Viotti *et al.*, 2023). However, the rate of meiotic error in mosaicisms identified on TE biopsy remains inconsistent across studies. Similar to our findings, a sequencing-based study reported that 3.7% (4/109) of whole-chromosome aneuploidies had intermediate CN changes, but none were found in segmental mosaicisms (De Witte *et al.*, 2024). Another study reported that 7.4% (8/108) of parental-biased mosaic CNVs were detected with a meiotic error (Zhang *et al.*, 2025). In contrast, a recent high-profile study revealed that one-third of 146 embryos initially diagnosed as mosaic by NGS were found to harbour meiotic errors in the SNP-based reanalysis (Popa *et al.*, 2025). This discrepancy likely reflects differences in the definition of mosaicism and technical bias across laboratory platforms. For example, Popa *et al.* employed a 30–80% deviation threshold for classifying mosaicism instead of the 30–70% threshold used in our study, which may overestimate mosaicism by misclassifying uniform meiotic aneuploidies as mosaic.

Mosaicism of mitotic origin in human blastocysts exhibits biased distribution patterns. Our results showed that chromosomal mosaicism, especially segmental mosaic losses, predominantly affected paternally derived chromosomes and coincided with the paternal bias previously observed in segmental aneuploidy in cleavage-stage embryos (Tsuiko *et al.*, 2021). In regards to the paternal bias toward segmental losses, a plausible explanation may be the lack of a DNA repair capacity in mature sperm cells that contributes to the susceptibility to DNA lesions at later stages of spermatogenesis. Upon fertilization, oocyte-derived factors may repair sperm DNA lesions via error-prone non-homologous end joining or homologous recombination (Marchetti *et al.*, 2007). However, if the oocyte fails to repair DNA double-strand breaks during the first mitosis, terminal loss of the acentric fragment may occur, predisposing it to postzygotic mitotic errors characterized by segmental losses (Tsuiko *et al.*, 2021).

In our study, factors associated with meiotic error in embryos with mosaic results were explored. Compared to embryos with low-level mosaicism, those with high-level mosaicism were associated with a 3.7-fold greater risk of meiotic error, providing a plausible explanation for reduced clinical pregnancy rates and increased miscarriage rates following transfers (Spinella *et al.*, 2018; Viotti *et al.*, 2021). A critical consideration is that high-level mosaicism of meiotic origin may actually reflect the misclassification of uniform meiotic aneuploidy due to technical noise. In addition, consistent with the results of a previous study (Zhang *et al.*, 2025), we observed that the maternal origin (93%) predominated in mosaicisms with a meiotic origin, suggesting an increased risk of meiotic errors in maternal-derived mosaic cases. Furthermore, we found that meiotic error rate was 9.6% for a maternal age ≥ 35 years. Therefore, we recommend PGT-AO for embryos exhibiting high-level mosaicism, those of maternal origin, or those from women of advanced maternal age. Our findings highlight the clinical value of PGT‑AO in interpreting mosaic biopsy results for risk assessment and embryo selection.

The overall clinical outcomes of METs of embryos with mitotic errors were comparable to those of euploid transfers in our study, which is consistent with the findings of a previous non-selective study (Capalbo *et al.*, 2021). Our results confirmed that embryo morphology was a significant factor influencing clinical pregnancy and LB/OP outcomes of blastocysts with mosaicism, which aligns with current consensus recommendations (De Rycke *et al.*, 2022). Compared with all concurrent EETs, our results showed that METs of embryos with mitotic errors were associated with a higher miscarriage rate, which is consistent with large-scale multicentre data (Viotti *et al.*, 2021, 2023). However, after adjusting for embryo quality, development day, and cycle-related confounders by PSM, no significant difference in the miscarriage rate was detected between the two groups. A plausible explanation is that mosaic embryos are typically selected for transfer only when no euploid embryos are available; accordingly, some patients undergoing MET may represent a poorer‑prognosis population. Notably, embryos with mosaicisms of meiotic origin were not transferred in our clinic due to ethical concerns. Therefore, the clinical outcomes of METs of embryos with meiotic errors remain unclear.

Concerns regarding the safety of METs for foetuses and neonates have been raised. In our prospective cohort, all live births following METs were confirmed to be euploid by karyotyping or CMA of prenatal samples. FISH analysis of postnatal and placental samples revealed only sporadic abnormal signals that were within the limits of technical variability. However, we confirmed PGT-A-identified mosaicisms in products of conception from two miscarriages and one termination case following MET. Notably, one case in which an embryo diagnosed with low-level mosaic monosomy 21 was transferred resulted in a foetus with uniform trisomy 21, and multisite placental biopsies confirmed mosaic trisomy 21. These findings challenge the previous assumption that mitotic-origin mosaicism generally exhibits a scattered cellular distribution rather than uniformity throughout all embryonic cells (Taylor *et al.*, 2014; Kim *et al.*, 2022). In alignment with these clinical observations, uniform aneuploidy across all single cells in a blastocyst with mosaicism of mitotic origin was identified in our single-cell verification. This phenomenon was consistent with previous reports of identical aneuploidy in all rebiopsies of an embryo with a mitotic error (Rana *et al.*, 2023). While embryos with mosaicisms of mitotic origin are currently available for transfer and typically yield favourable outcomes (Currie *et al.*, 2022; De Rycke *et al.*, 2022), they still carry non-negligible risks. Therefore, comprehensive genetic counselling is essential for patients prior to embryo transfer (Leigh *et al.*, 2022).

The strengths of this study include the following. First, we refined and validated a comprehensive SNP-based approach by combining a BAF analysis with CN quantification for the simultaneous analysis of the origin and quantification of mosaicism, facilitating the accurate diagnosis of embryos with mosaic results. Second, the parental and cell-division origins of blastocysts with mosaicisms were identified in a larger cohort than those in previous reports. Moreover, pregnancy outcomes following MET of embryos with mitotic origin were prospectively evaluated and compared with following transfer of euploid controls. Finally, blastocysts identified as mosaic on biopsy were dissected at a single-cell resolution to determine the relationship between the origin of chromosome abnormalities and mosaic composition.

Our study had several limitations. First, the PGT-A diagnosis based on a single biopsy result has inherent limitations, since a single TE biopsy may not represent the chromosomal status of the whole embryo or ICM. Interpreting mosaicism based on intermediate CN values is technically constrained, as intrinsic technical artifacts cannot be definitively distinguished from genuine mosaicism using our PGT‑AO platform. Second, we excluded mosaic uniparental disomy and complex mosaicism involving >3 chromosomes. Non-recombinant meiotic errors cannot be distinguished from mitotic errors using the current methods, highlighting the need for optimized diagnostic approaches. Third, the MET cohort was limited, with few cases undergoing prenatal and postnatal genetic validation. Only a small number of embryos with high-level mosaicisms were included in the transfer cohort, potentially limiting our ability to evaluate their clinical impact. Further investigations in a large-scale and multicentre cohort are warranted. Last but not the least, single-cell verification was conducted in only a small subset of mosaic embryos, and CNVs smaller than 10 Mb across the genome were underestimated due to technical limitations at the single-cell level.

In conclusion, integrated mosaicism quantification and PGT-AO analyses revealed a low prevalence of meiotic errors in embryos classified as mosaic on biopsy. We recommend PGT-AO for embryos with high-level mosaicism or from women of advanced maternal age. Although a low risk was observed in METs with mitotic error, a longitudinal genetic assessment and long-term offspring follow-up after MET are necessary.

## Supplementary Material

hoag067_Supplementary_Data

## Data Availability

Due to the individual privacy of the patient’s health information, the datasets supporting the study have not been deposited in a public repository but are available from the corresponding author on reasonable request.
