# Supplementary material for: Risk factors associated with meiotic errors in blastocysts with mosaic results on biopsy
Source: Hum Reprod Open. 2026 Jul 24;2026(3):hoag067. doi: 10.1093/hropen/hoag067 (PMC13461220; doi:10.1093/hropen/hoag067)
Supplement: Supplementary file 1 [file hoag067_supplementary_data.zip › HRO-25-0717 - Supplementary Fig. S1 Final.pdf]

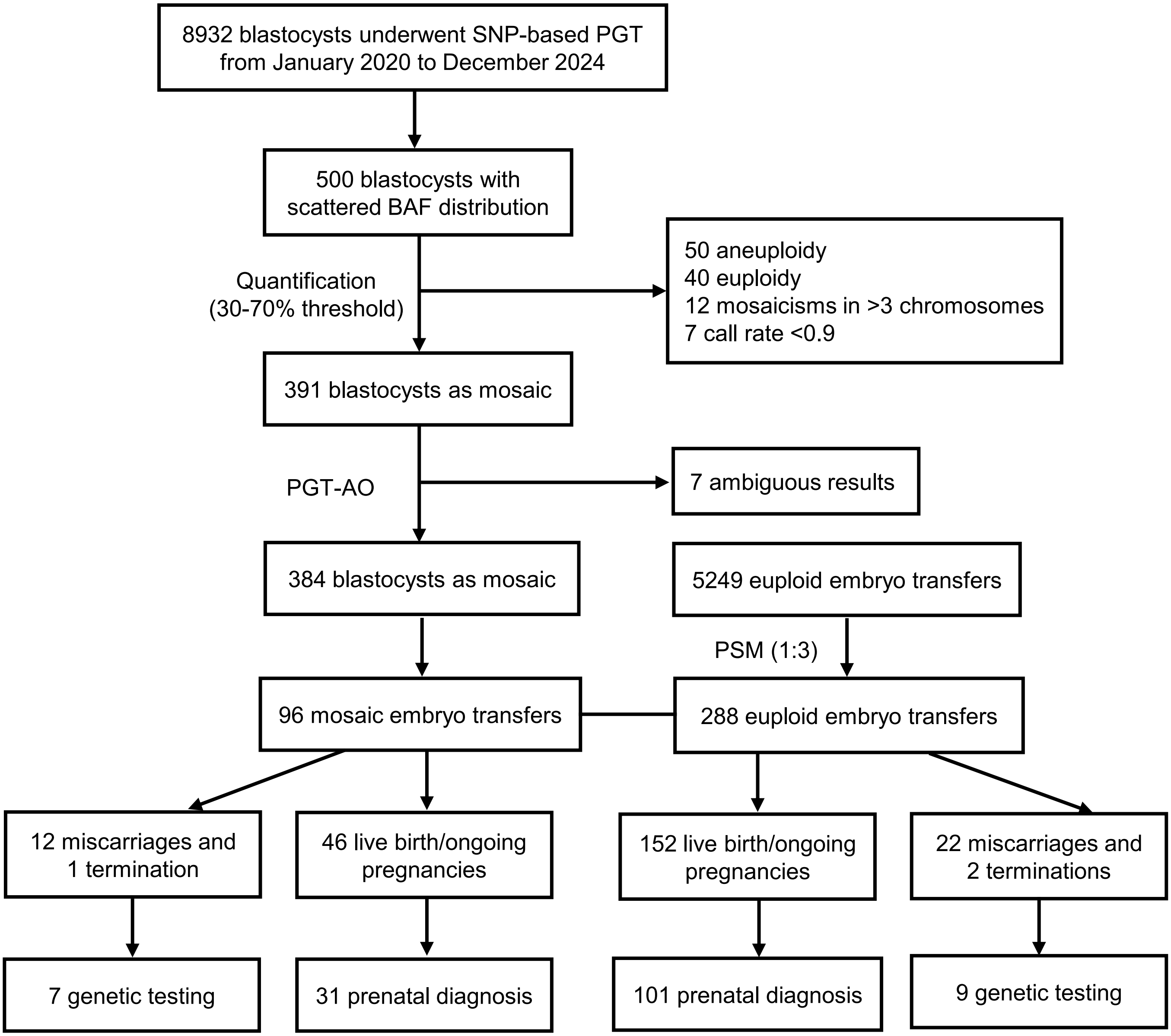

Supplementary Fig. S1 Flowchart of embryo cohort.
BAF, B allele frequency; PGT, preimplantation genetic testing; PGT-AO: preimplantation genetic testing for aneuploidy origin; PSM, propensity score matching; SNP, single nucleotide polymorphism.
