# Supplementary material for: Risk factors associated with meiotic errors in blastocysts with mosaic results on biopsy
Source: Hum Reprod Open. 2026 Jul 24;2026(3):hoag067. doi: 10.1093/hropen/hoag067 (PMC13461220; doi:10.1093/hropen/hoag067)
Supplement: Supplementary file 1 [file hoag067_supplementary_data.zip › Supplementary Fig. S2.pdf]

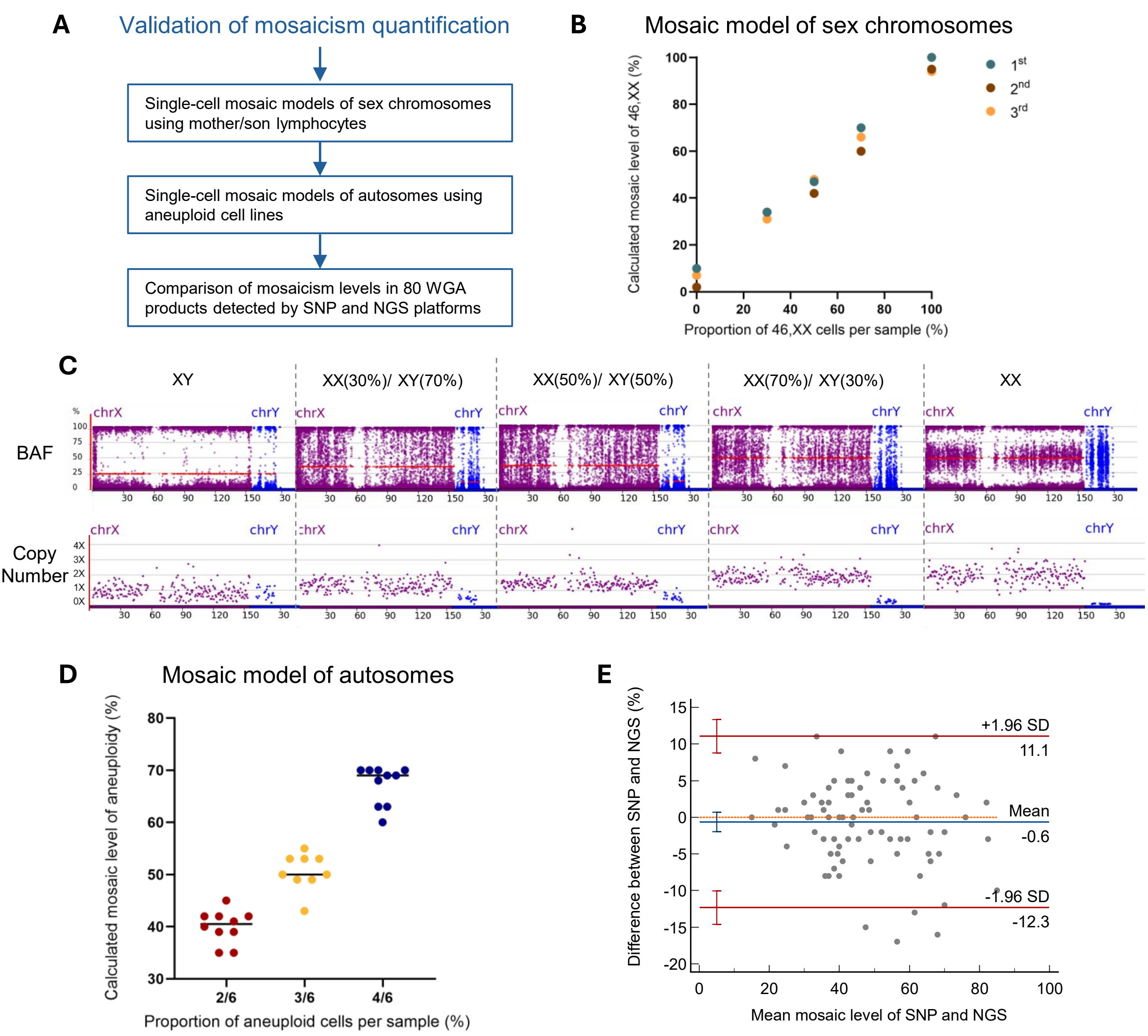

Supplementary Fig. S2 Validation of SNP-based mosaicism quantification.
(A) Three approaches were used for validation of mosaicism quantification. (B) Comparison of the calculated mosaic level of 46, XX cells with the actual mixing proportion of mother cells using mother/son lymphocytes in three separate experiments, observing a <10% deviation. (C) B allele frequency value and copy number profile in mosaic models using mother/son lymphocyte mixtures. (D) Comparison of the calculated mosaic level of targeted aneuploid cells with the actual mixing proportion of aneuploid cells in the mosaic models of autosomes. Experiments were repeated ten times. Proportion 2/6 referred to the presence of two aneuploid cells from an aneuploid cell line in a six-cell sample. (E) Bland-Altman plots showing the differences in mosaic levels between SNP and NGS methods in amplified products from 80 blastocysts. The x-axis represents the mean of the mosaic levels calculated by two methods, and the y-axis shows the difference between them (the mosaic level by the SNP method minus that by the NGS method). The blue line indicates the mean difference and 95% confidence interval (CI), and red lines represent limits of agreement and 95% CI. BAF, B allele frequency; NGS, next-generation sequencing; SNP, single nucleotide polymorphism.
