# Supplementary material for: Risk factors associated with meiotic errors in blastocysts with mosaic results on biopsy
Source: Hum Reprod Open. 2026 Jul 24;2026(3):hoag067. doi: 10.1093/hropen/hoag067 (PMC13461220; doi:10.1093/hropen/hoag067)
Supplement: Supplementary file 1 [file hoag067_supplementary_data.zip › Supplementary Fig. S3.pdf]

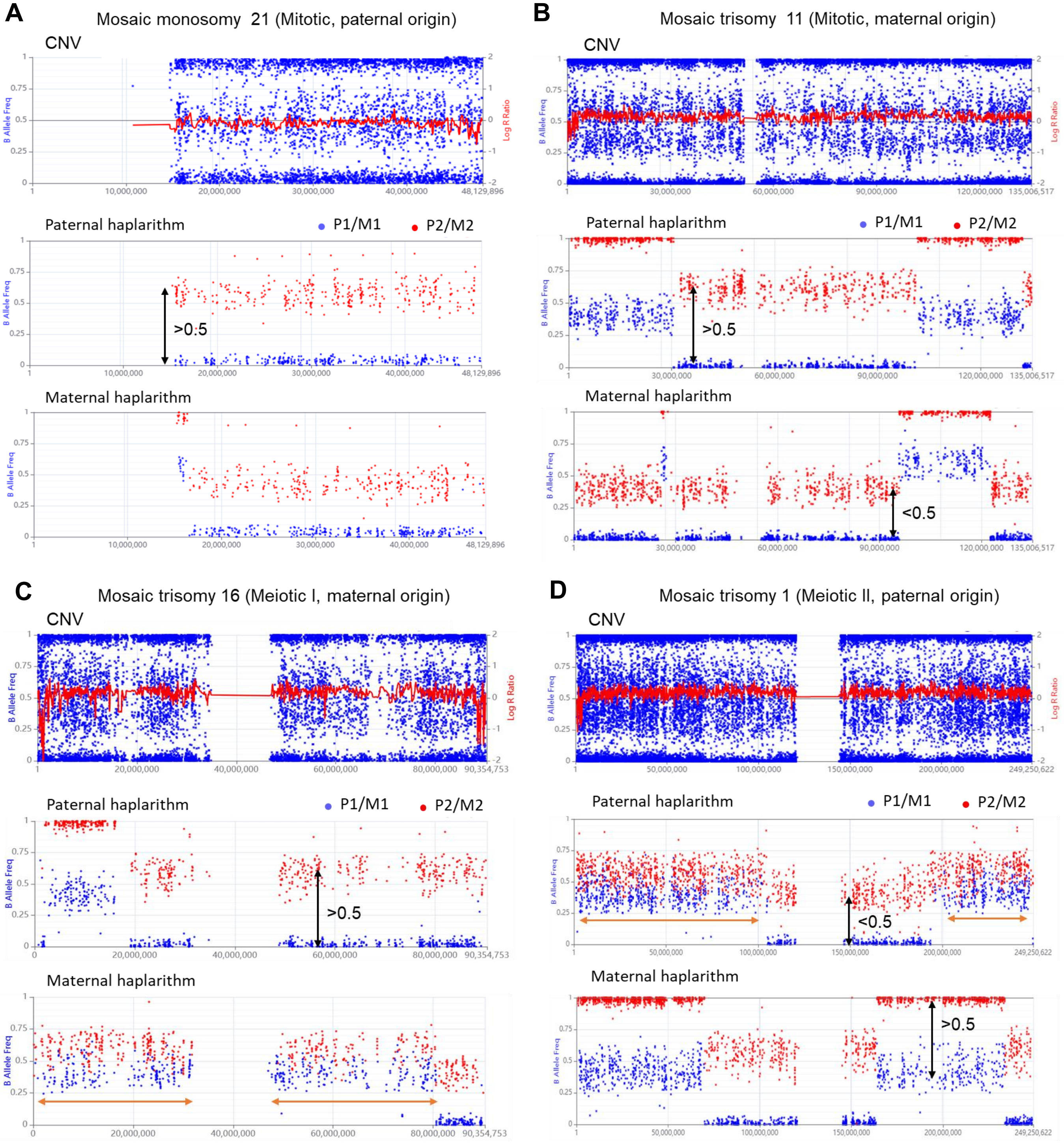

Supplementary Fig. S3 Distinct haplarithm patterns for chromosomal mosaicism.
(A) Mitotic mosaic monosomy. The CNV plot showed mosaic monosomy 21. BAF values were distributed between 0 and 0.5 or 0.5 and 1 across the whole chromosome, indicating the presence of heterozygous SNPs originated from mitotic origin. An increased distance over 0.5 was observed in the paternal informative SNPs, indicating a chromosomal loss of paternal origin. (B) Mitotic mosaic trisomy. The CNV plot showed mosaic trisomy 11. Maternal haplarithm profiles showed that distance between M1 and M2 was less than 0.5, indicating a chromosomal gain of maternal origin. Maternal informative SNPs contained BAF values of 0 or 1 across the whole chromosome, indicating single parental homolog (SPH) patterns originated from a maternal mitotic error. (C) Meiotic I mosaic trisomy. The CNV plot showed mosaic trisomy 16. Maternal haplarithm profiles showed both parental homologs (BPH) patterns with BAF values of M1 and M2 around 0.5. Meiotic I errors were identified by the presence of BPH in the pericentromeric region. The distance (<0.5) between M1 and M2 implied chromosomal gains of maternal origin. (D) Meiotic II mosaic trisomy. The CNV plot indicated mosaic trisomy 1. Paternal haplarithm profiles showed BPH patterns, with BAF values of P1 and P2 around 0.5. Meiotic II errors were identified by the presence of BPH regions at the ends of chromosomes. The distance (<0.5) between M1 and M2 implied chromosomal gains of maternal origin.
Black double-headed arrows denoted the distance of the informative SNPs. Orange double-headed arrows denoted BPH regions. CNV, copy number variant.
