# Supplementary material for: Risk factors associated with meiotic errors in blastocysts with mosaic results on biopsy
Source: Hum Reprod Open. 2026 Jul 24;2026(3):hoag067. doi: 10.1093/hropen/hoag067 (PMC13461220; doi:10.1093/hropen/hoag067)
Supplement: Supplementary file 1 [file hoag067_supplementary_data.zip › Supplementary Fig. S4.pdf]

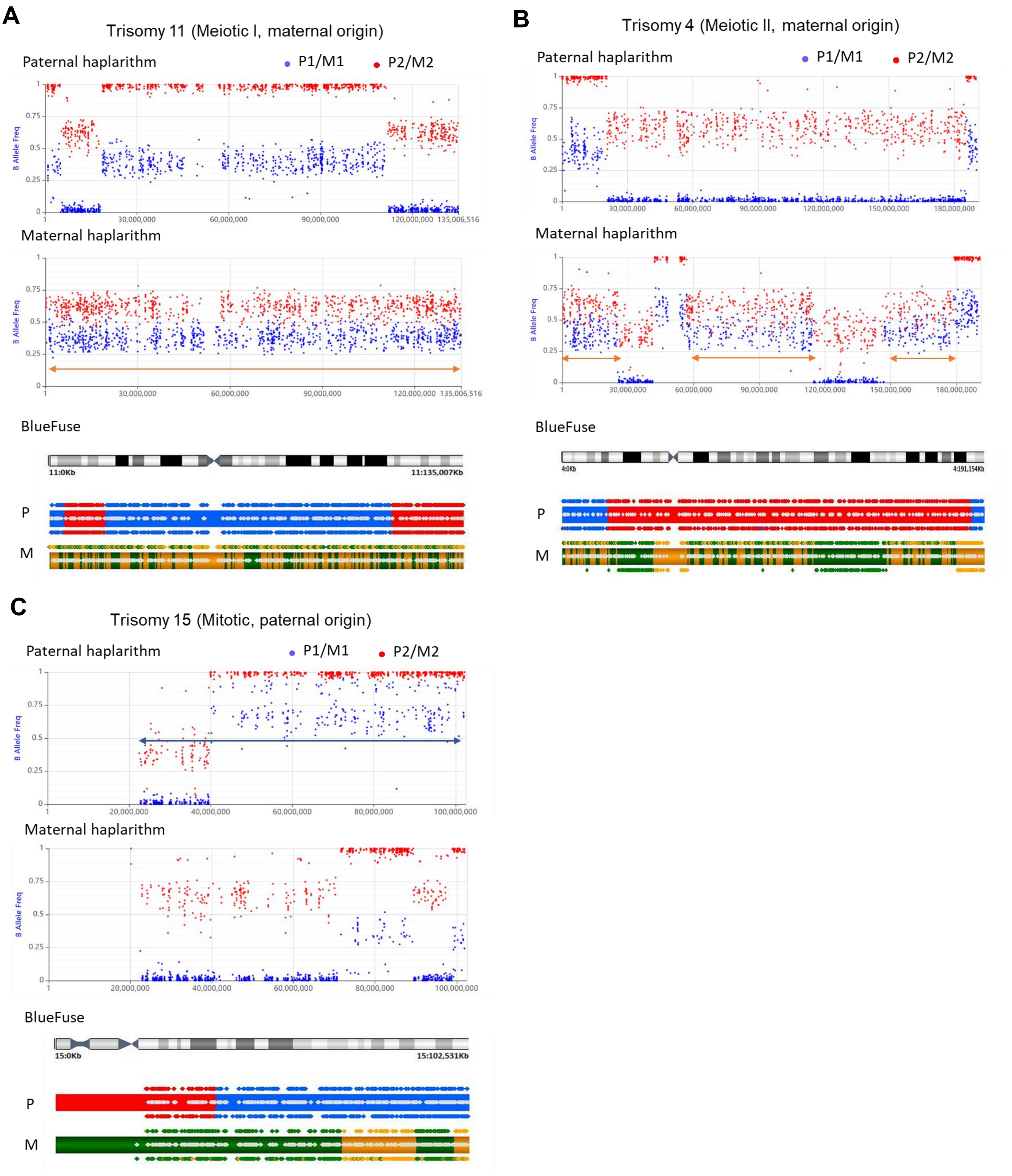

Supplementary Fig. S4 Performance of origin analysis in trisomy between PGT-AO and BlueFuse platform.

(A) Trisomy 11 originating from maternal meiotic I error. Maternal haplarithm profiles showed both parental homologs (BPH) patterns across the pericentromeric region. Haploblock chart in BlueFuse showing maternal haplotypes with only informative SNPs, an indication of BPH. (B) Trisomy 4 originating from maternal meiotic II error. Maternal haplarithm profiles showed BPH patterns distributed away from the centromeric region. Haploblock chart showing maternal haplotypes with only informative SNPs, an indication of BPH. (C) Trisomy 15 originating from paternal mitotic error. Single parental homolog (SPH) patterns presented in paternal haplarithm, which were not visible on the haploblock chart in BlueFuse.

Orange double-headed arrows denoted BPH regions. Blue double-headed arrows denoted SPH regions. In the haploblock chart of the BlueFuse, maternal chromosomes are visualized in green and yellow and paternal in blue and red colors.

M, maternal chromosome; P, paternal chromosome.
