# Supplementary material for: Risk factors associated with meiotic errors in blastocysts with mosaic results on biopsy
Source: Hum Reprod Open. 2026 Jul 24;2026(3):hoag067. doi: 10.1093/hropen/hoag067 (PMC13461220; doi:10.1093/hropen/hoag067)
Supplement: Supplementary file 1 [file hoag067_supplementary_data.zip › Supplementary Fig. S5.pdf]

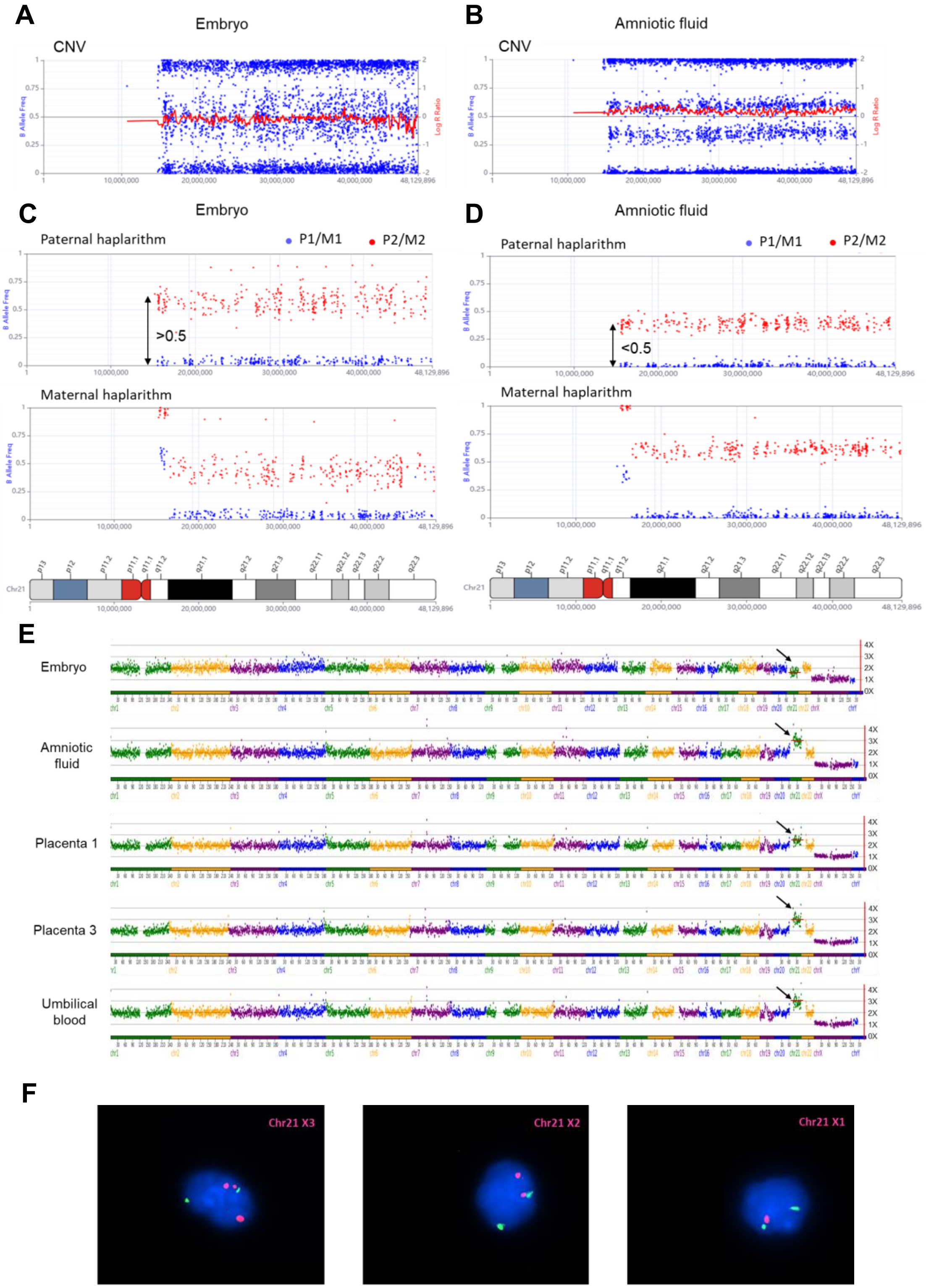

G

Sample	Karyotype in SNP array	FISH analysis			
		Cells with one chr21 signal	Cells with two chr21 signals	Cells with three chr21 signals	Number of analyzed cells
Umbilical blood	+21	0	0	100	100
Placenta 1	mos +21 (41%)	2	66	32	100
Placenta 2	mos +21 (31%)	1	73	26	100
Placenta 3	+21	0	0	100	100
Placenta 4	+21	0	0	100	100
Placenta 5	mos +21 (48%)	3	59	38	100

Supplementary Fig. S5 A case with trisomy 21 pregnancy following transfer of a blastocyst with low-level mosaicism.

(A) PGT-A result of clinical TE biopsy by SNP array showing mosaic monosomy 21 (35%). (B) Amniotic fluid detected by SNP array showed trisomy 21. (C) Parental haplarithm profiles in chromosome 21 showed paternal deletion with mitotic error in the embryo. An increased distance over 0.5 was observed in the paternal informative SNPs, indicating a low proportion of paternal chromosome. (D) Paternal duplication with mitotic error in the amniotic fluid. A decreased distance less than 0.5 was observed among paternal informative SNPs, indicating a high proportion of paternal chromosome. BAF values containing 0 or 1 across the whole chromosome indicated the single parental homolog region originated form a mitotic error. Black double-headed arrows denoted the distance of the informative SNPs. (E) NGS profiles showed the karyotypes in the embryo (mos -21, 35%), amniotic fluid (+21), placenta 1 (mos +21, 41%), placenta 3 (+21), and umbilical blood (+21), respectively. Black arrows denoted abnormal copy number in chromosome 21. (F) FISH images showed cells with 1 to 3 signals of chromosome 21 among cells in different site of placenta tissues. The 21qter subtelomere labeled with red signals, and 3qter subtelomere probe with green signals was set as a control. Images were captured at 40×magnification. (G) SNP and FISH analysis in umbilical blood and 5 sites of placental tissues.

FISH, fluorescent *in situ* hybridization; mos, mosaic; NGS, next-generation sequencing; PGT, preimplantation genetic testing; SNP, single nucleotide polymorphism; TE, trophectoderm.
