# Supplementary material for: Risk factors associated with meiotic errors in blastocysts with mosaic results on biopsy
Source: Hum Reprod Open. 2026 Jul 24;2026(3):hoag067. doi: 10.1093/hropen/hoag067 (PMC13461220; doi:10.1093/hropen/hoag067)
Supplement: Supplementary file 1 [file hoag067_supplementary_data.zip › Supplementary Fig. S6.pdf]

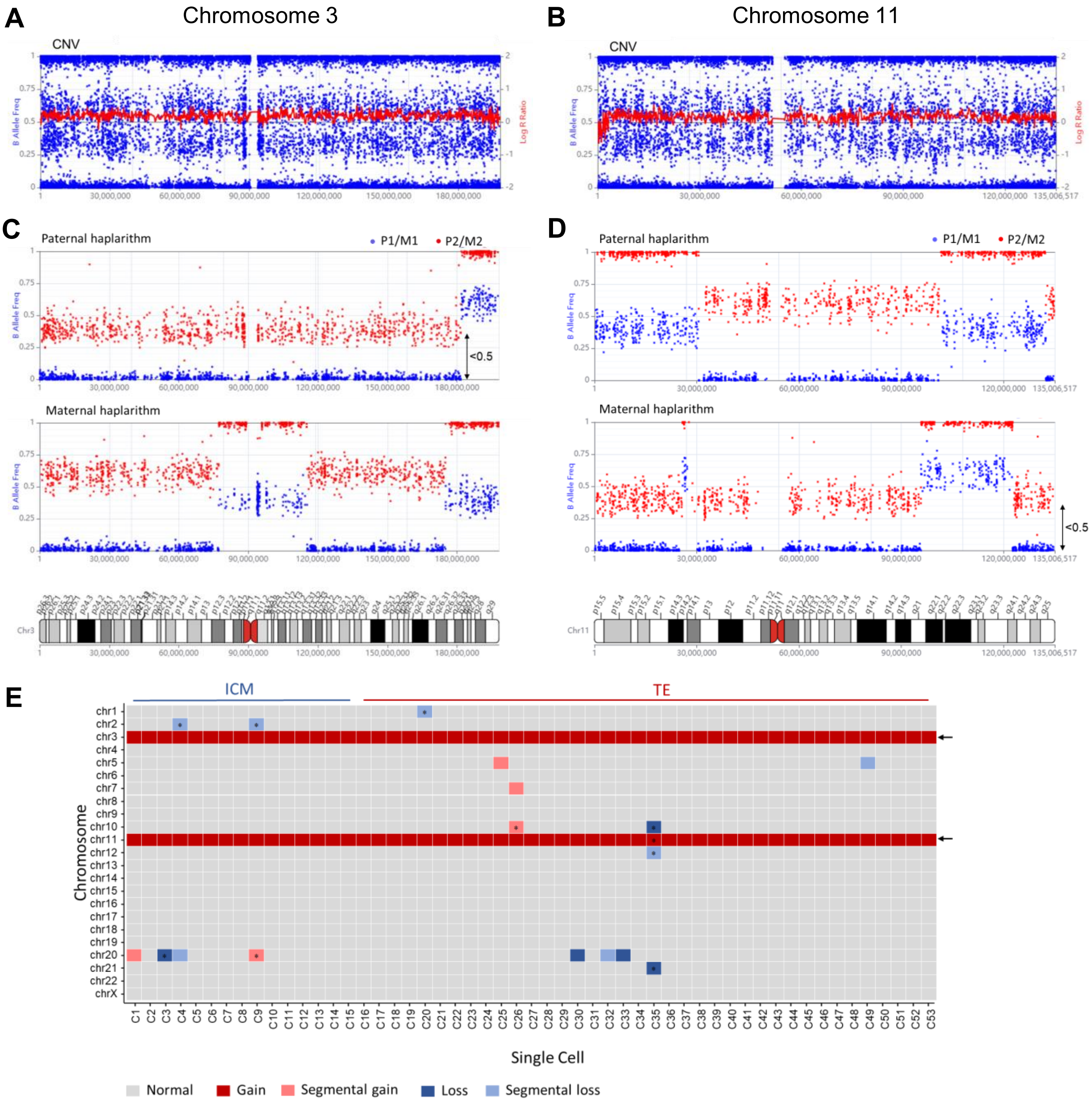

Supplementary Fig. S6 Single-cell results of uniform aneuploidy in a blastocyst initially diagnosed with a high-level mosaicism of mitotic error (Embryo 4).

BAF values and Log R Ratio results of initial TE biopsy showing trisomy 3 (A) and mosaic trisomy 11 (B). Parental haplarithm profiles of initial TE biopsy showed paternal mitotic origin in trisomy 3 (C) and maternal mitotic origin in mosaic trisomy 11 (D). For trisomies, BAF values of informative SNPs from abnormal parental origin were positioned on the same side of the 0.5 axis (a decreased distance less than 0.5). BAF values containing 0 or 1 distributed in the whole chromosome indicated the single parental homolog region originated form a mitotic error. Black double-headed arrows represented the distance of the informative SNPs. (E) Heatmap showing copy number analysis profiles of single cells (n=53). Black arrows denoted the initial mosaic chromosomes detected in TE biopsy sample.

* indicated an intermediate copy number signal result by NGS.

C, cell; ICM, inner cell mass; TE, trophectoderm.
