# Supplementary material for: Risk factors associated with meiotic errors in blastocysts with mosaic results on biopsy
Source: Hum Reprod Open. 2026 Jul 24;2026(3):hoag067. doi: 10.1093/hropen/hoag067 (PMC13461220; doi:10.1093/hropen/hoag067)
Supplement: Supplementary file 1 [file hoag067_supplementary_data.zip › Supplementary Fig. S7.pdf]

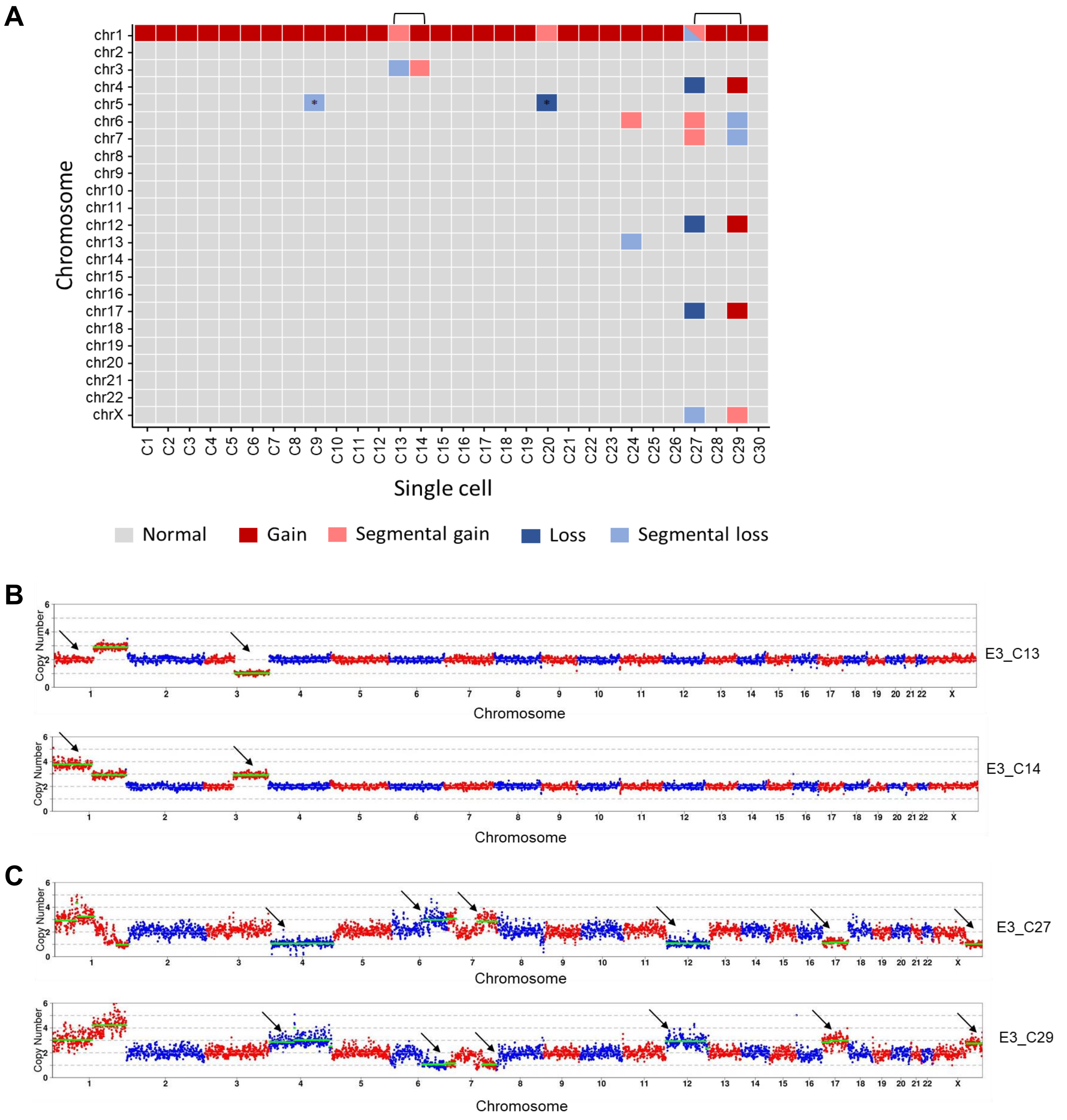

Supplementary Fig. S7 Reciprocal events revealed by the single-cell sequencing.
Embryo 3 was diagnosed as "mos +1" in PGT-A result. (A) Heatmap showing copy number analysis profiles of ICM cells (n=30) in the Embryo 3. Two pairs of cells displayed reciprocal chromosomal gains and losses on the same chromosome. (B) NGS results showing E3_C13 was observed with dup1q21.1q44 ×3 and del3q11.1q29, while E3_C14 was observed with dup1p36.33→q21.1 ×4, dup1q21.1q44 ×3 and dup3q11.1q29 ×3. (C) NGS results showing E3_C27 and E3_C29 were observed with reciprocal chromosomal gains and losses on chromosome 4, 6, 7, 12, 17 and X. Black arrows denoted the reciprocal chromosomes.

* indicating an intermediate copy number signal result by NGS.

C, cell; del, deletion; dup, duplication; E, Embryo.
