# Supplementary material for: Risk factors associated with meiotic errors in blastocysts with mosaic results on biopsy
Source: Hum Reprod Open. 2026 Jul 24;2026(3):hoag067. doi: 10.1093/hropen/hoag067 (PMC13461220; doi:10.1093/hropen/hoag067)
Supplement: Supplementary file 1 [file hoag067_supplementary_data.zip › Supplementary Table S1.docx]

**Supplementary Table S1 Comparison in determining the origin of chromosomal abnormalities between PGT-AO and BlueFuse platform.**

| **Chromosomal abnormality** | **Cell-division origin** | | **No. of embryo** | **Concordance rate in cell-division origin** | **Concordance rate in parental origin** |
| --- | --- | --- | --- | --- | --- |
|  | **PGT-AO** | **BlueFuse** |  |  |  |
| Unbalanced segregations from translocation carriers | Meiotic I | Meiotic I | 15 | 100% | 100% |
| Trisomy | Meiotic I | Meiotic I | 34 | 100% | 100% |
|  | Meiotic II | Meiotic II | 25 | 100% | 100% |
|  | Mitotic | Unidentified ^a^ | 10 | Unidentified | Unidentified |

^a^ As single parental homolog patterns in the chromosomal gain cannot be visible in the haploblock chart on BlueFuse, the parental origin of mitotic-error trisomies consequently cannot be determined (Kubicek *et al.*, 2019).

PGT-AO, preimplantation genetic testing for aneuploidy origin.
