# Supplementary material for: Risk factors associated with meiotic errors in blastocysts with mosaic results on biopsy
Source: Hum Reprod Open. 2026 Jul 24;2026(3):hoag067. doi: 10.1093/hropen/hoag067 (PMC13461220; doi:10.1093/hropen/hoag067)
Supplement: Supplementary file 1 [file hoag067_supplementary_data.zip › Supplementary Table S2.docx]

**Supplementary Table S2 Mosaicism with meiotic error identified in the retrospective cohort**

| **Embryo** | **PGT-A results** | **Chromosome** | **Mosaicism level (%)** | **Parental origin** | **Cell-division origin** |
| --- | --- | --- | --- | --- | --- |
| E2_1 | mos +1 | 1 | 64 | Pat | Meiotic II |
| E2_2 | mos +1q11 | 1 | 35 | Mat | Meiotic II |
| E2_3 | mos +2 | 2 | 32 | Mat | Meiotic II |
| E2_4 | mos +9 | 9 | 34 | Mat | Meiotic II |
| E2_5 | mos dup 9p24.3-q34.11 | 9 | 52 | Mat | Meiotic II |
| E2_6 | mos +11p | 11 | 60 | Mat | Meiotic II |
| E2_7 | mos +16 | 16 | 64 | Mat | Meiotic I |
| E2_8 | mos dup 17p11.2 | 17 | 52 | Mat | Meiotic II |
| E2_9 | mos +20 | 20 | 37 | Mat | Meiotic II |
| E2_10 | mos +22 | 22 | 64 | Mat | Meiotic II |
| E2_11 | mos +22 | 22 | 65 | Mat | Meiotic I |
| E2_12 | mos +22 | 22 | 66 | Mat | Meiotic II |
| E2_13 | mos +22 | 22 | 67 | Mat | Meiotic I |
| E2_14 | mos dup 1q, mos +X | 1 | 46 | Pat | Mitotic ^a^ |
|  |  | X | 35 | Mat | Meiotic II |
| E2_15 | mos dup 11p11.2, mos +22 | 11 | 63 | Pat | Mitotic ^a^ |
|  |  | 22 | 43 | Mat | Meiotic II |

^a^ Single parental homologous signatures were present in mosaic chromosomal gains, indicating mitotic errors.

dup, duplication; Mat, maternal; mos, mosaic; Pat, paternal; PGT-A: preimplantation genetic testing for aneuploidy.
