# Supplementary material for: Risk factors associated with meiotic errors in blastocysts with mosaic results on biopsy
Source: Hum Reprod Open. 2026 Jul 24;2026(3):hoag067. doi: 10.1093/hropen/hoag067 (PMC13461220; doi:10.1093/hropen/hoag067)
Supplement: Supplementary file 1 [file hoag067_supplementary_data.zip › Supplementary Table S3.docx]

**Supplementary Table S3 Baseline characteristics before and after 1:3 propensity score matching between mosaic embryo transfers with mitotic origin and euploid embryo transfers.**

| **Characteristics** | **Before PSM** | | | |  | **After PSM** | | | |
| --- | --- | --- | --- | --- | --- | --- | --- | --- | --- |
|  | **MET (n=96)** | **EET (=5249)** | ***P* value** | **SMD** |  | **MET (n=96)** | **EET (=288)** | ***P* value** | **SMD** |
| Maternal age (years) | 30 (29, 34) | 31 (28, 34) | 0.636 | -0.083 |  | 30 (29, 34) | 30 (28, 33) | 0.320 | 0.132 |
| Maternal BMI (kg/m^2^) | 21.28 (19.83, 22.91) | 20.96 (19.52, 22.96) | 0.568 | 0.070 |  | 21.28 (19.83, 22.91) | 20.82 (19.51, 23.11) | 0.468 | 0.074 |
| Basal FSH (IU/L) | 5.64 (4.63, 6.71) | 5.50 (4.76, 6.39) | 0.558 | 0.027 |  | 5.64 (4.63, 6.71) | 5.55(4.83, 6.45) | 0.977 | -0.057 |
| Gravidity, n (%) | - | - | 0.533 | - |  | - | - | 0.676 | - |
| 0 | 21 (21.88) | 1015 (19.34) | - | 0.061 |  | 21 (21.88) | 69 (23.96) | - | -0.050 |
| ≥1 | 75 (78.12) | 4234 (80.66) | - | -0.061 |  | 75 (78.12) | 219 (76.04) | - | 0.050 |
| PGT indication, n (%) | - | - | **<0.001** | - |  | - | - | 0.746 | - |
| PGT-SR | 63 (65.62) | 1901 (36.22) | - | 0.619 |  | 63 (65.62) | 185 (64.24) | - | 0.029 |
| PGT-M+PGT-A | 31 (32.29) | 2321 (44.22) | - | -0.255 |  | 31 (32.29) | 99 (34.38) | - | -0.045 |
| PGT-A | 2 (2.08) | 1027 (19.57) | - | -1.224 |  | 2 (2.08) | 4 (1.39) | - | 0.049 |
| Ovarian stimulation protocol, n (%) | - | - | 0.592 | - |  | - | - | 0.723 | - |
| GnRH-a protocol | 52 (54.17) | 3075 (58.58) | - | -0.089 |  | 52 (54.17) | 150 (52.08) | - | 0.042 |
| GnRH-ant protocol | 44 (45.83) | 2100 (40.01) | - | 0.117 |  | 44 (45.83) | 138 (47.92) | - | -0.042 |
| Minimal stimulation | 0 | 59 (1.12) | - | -0.108 |  | 0 | 0 | - | - |
| Others | 0 | 15 (0.29) | - | -0.054 |  | 0 | 0 | - | - |
| No. of retrieved oocyte | 15 (12, 22.25) | 18 (12, 23) | 0.171 | -0.071 |  | 15 (12, 22.25) | 17 (12, 22) | 0.584 | -0.024 |
| No. of biopsied blastocyst | 6 (4, 8) | 7 (4, 10) | **0.002** | -0.383 |  | 6 (4, 8) | 5 (3.75, 9) | 0.955 | -0.038 |
| Prior transfer cycles, n (%) | - | - | 0.604 | - |  | - | - | 0.567 | - |
| <2 cycles | 83 (86.46) | 4437 (84.53) | **-** | 0.056 |  | 83 (86.46) | 242 (84.03) | - | 0.071 |
| ≥2 cycles | 13 (13.54) | 812 (15.47) | - | -0.056 |  | 13 (13.54) | 46 (15.97) | **-** | -0.071 |
| Endometrial preparation, n (%) | - | - | 0.363 | - |  | - | **-** | 0.551 | **-** |
| Natural cycle | 38 (39.58) | 1776 (33.84) | - | 0.118 |  | 38 (39.58) | 105 (36.46) | **-** | 0.064 |
| HRT | 52 (54.17) | 2980 (56.77) | - | -0.052 |  | 52 (54.17) | 171 (59.38) | **-** | -0.105 |
| Induced ovulation | 6 (6.25) | 493 (9.39) | - | -0.130 |  | 6 (6.25) | 12 (4.17) | **-** | 0.086 |
| Developmental speed, n (%) | - | - | **<0.001** | - |  | - | - | 0.899 | **-** |
| Day 5 | 28 (29.17) | 2713 (51.69) | - | -0.495 |  | 28 (29.17) | 79 (27.43) | - | 0.038 |
| Day 6 | 59 (61.46) | 2211 (42.12) | - | 0.397 |  | 59 (61.46) | 178 (61.81) | - | -0.007 |
| Day 7 | 9 (9.38) | 325 (6.19) | - | 0.109 |  | 9 (9.38) | 31 (10.76) | - | -0.048 |
| Blastocyst quality, n (%) | - | - | **0.039** | - |  | - | - | 0.966 | **-** |
| Good | 28 (29.17) | 2114 (40.27) | - | -0.244 |  | 28 (29.17) | 80 (27.78) | - | 0.031 |
| Average | 48 (50.00) | 2404 (45.80) | - | 0.084 |  | 48 (50.00) | 147 (51.04) | - | -0.021 |
| Poor | 20 (20.83) | 731 (13.93) | - | 0.170 |  | 20 (20.83) | 61 (21.18) | - | -0.009 |

Maternal age, maternal BMI, and basal FSH were tested at the oocyte retrieval cycle.

Skewed quantitative variables were described by median (interquartile) and compared using Mann-Whiney U Test. Categorical variables were described by number (percentage) and analyzed using Chi-square test or Fisher’s exact test. Bolded results were statistically significant (*P* value < 0.05).

BMI, body mass index; EET, euploid embryo transfer; FSH, follicle stimulating hormone; GnRH-a, gonadotrophin-releasing hormone agonist; GnRH-ant, gonadotrophin-releasing hormone antagonist; HRT, hormone replacement therapy; MET, mosaic embryo transfer; PGT, preimplantation genetic testing; PGT-A, PGT for aneuploidy; PGT-M, PGT for monogenic disorders; PGT-SR, PGT for structural rearrangements; PSM, propensity score matching; SMD, standardized mean difference.
