# Supplementary material for: Risk factors associated with meiotic errors in blastocysts with mosaic results on biopsy
Source: Hum Reprod Open. 2026 Jul 24;2026(3):hoag067. doi: 10.1093/hropen/hoag067 (PMC13461220; doi:10.1093/hropen/hoag067)
Supplement: Supplementary file 1 [file hoag067_supplementary_data.zip › Supplementary Table S4.docx]

**Supplementary Table S4 Multivariable** **logistic regression analysis of risk factors related to** **pregnancy outcomes following mosaic embryo transfers with mitotic origin**

| **Characteristics** | **Clinical pregnancy** | |  | **Live birth/ongoing pregnancy** | |  | **Miscarriage** | |
| --- | --- | --- | --- | --- | --- | --- | --- | --- |
|  | ***P* value** | **OR (95% CI)** |  | ***P* value** | **OR (95% CI)** |  | ***P* value** | **OR (95% CI)** |
| Maternal age ^a^ | 0.140 | **-** |  | 0.062 | **-** |  | 0.964 | **-** |
| Maternal BMI ^a^ | 0.738 | **-** |  | 0.233 | **-** |  | 0.961 | **-** |
| Basal FSH ^a^ | 0.076 | - |  | 0.368 | - |  | 0.989 | - |
| Gravidity | 0.506 | **-** |  | 0.281 | **-** |  | 0.961 | **-** |
| 0 | Reference | - |  | Reference | - |  | Reference | - |
| ≥1 | 0.506 | - |  | 0.281 | - |  | 0.961 | - |
| No. of retrieved oocyte | 0.501 | - |  | 0.146 | - |  | 0.967 | - |
| No. of biopsied blastocyst | 0.652 | - |  | 0.697 | - |  | 0.966 | - |
| Prior transfer cycles | 0.613 | **-** |  | 0.465 | **-** |  | 0.964 | **-** |
| <2 cycles | Reference | - |  | Reference | - |  | Reference | - |
| ≥2 cycles | 0.613 | - |  | 0.465 | - |  | 0.964 | - |
| Endometrial preparation | 0.120 | - |  | 0.468 | - |  | 0.999 | - |
| Natural cycle | Reference | **-** |  | Reference | **-** |  | Reference | **-** |
| HRT | 0.831 | **-** |  | 0.279 | - |  | 0.962 | - |
| Induced ovulation | **0.046** | **0.076 (0.006-0.952)** |  | 0.374 | - |  | 0.985 | - |
| Developmental day | **0.022** | - |  | 0.799 | - |  | 1.000 | - |
| Day 5 | Reference | **-** |  | Reference | - |  | Reference | - |
| Day 6 | 0.952 | - |  | 0.503 | - |  | 0.991 | **-** |
| Day 7 | **0.014** | **0.015 (0.000-0.428)** |  | 0.997 | - |  | 0.980 | - |
| Blastocyst quality ^b^ | **0.001** | - |  | **0.015** | - |  | 0.999 | **-** |
| Good | Reference | - |  | Reference | - |  | Reference | **-** |
| Average | 0.300 | **-** |  | 0.111 | - |  | 0.962 | - |
| Poor | **0.001** | **0.021 (0.002-0.207)** |  | **0.004** | **0.053 (0.007-0.387)** |  | 0.964 | **-** |
| Mosaicism level | 0.612 | - |  | 0.992 | **-** |  | 0.984 | - |
| Low-level | Reference | - |  | Reference | **-** |  | Reference | - |
| High-level | 0.612 | **-** |  | 0.992 | - |  | 0.984 | - |
| No. of mosaic chromosome | 0.054 | - |  | 0.369 | **-** |  | 0.980 | - |
| 1 | Reference | - |  | Reference | - |  | Reference | **-** |
| ≥2 | 0.054 | - |  | 0.369 | **-** |  | 0.980 | - |
| Copy number | 0.961 | **-** |  | 0.963 | **-** |  | 0.999 | - |
| Loss | Reference | **-** |  | Reference | - |  | Reference | - |
| Gain | 0.778 | **-** |  | 0.783 | **-** |  | 0.999 | **-** |
| Both loss and gain | 0.999 | **-** |  | 0.999 | - |  | - | - |
| Mosaic chromosome length | 0.892 | **-** |  | 0.960 | - |  | 0.999 | - |
| Segmental | Reference | - |  | Reference | - |  | Reference | - |
| Whole chromosome | 0.772 | - |  | 0.774 | - |  | 0.961 | - |
| Segmental and whole chromosome | 0.681 | - |  | 0.998 | **-** |  | 0.986 | **-** |
| Parental origin | 0.472 | - |  | 0.904 | - |  | 0.961 | - |
| Paternal origin | Reference | - |  | Reference | - |  | Reference |  |
| Maternal origin | 0.524 | - |  | 0.653 | - |  | 0.961 | - |
| Both origin | 0.279 | - |  | 0.998 | **-** |  | - | - |

Multivariable logistic regression was used to assess risk factors for pregnancy outcomes following mosaic embryo transfers with mitotic origin, with all covariates entered simultaneously into the model. Results are shown as odds ratios (OR) with 95% confidence intervals (CI). A *P* value < 0.05 was considered statistically significant. *P* values with statistical significance and odds ratios were marked in bold.

^a^ Characteristics were tested at the oocyte retrieval cycle.

^b^ Blastocysts quality was classified based on their morphologic grading using Gardner’s grading system: good (≥3AA, AB and BA), average (≥3 BB, AC and CA) and poor (≥3 BC, CB and CC).

BMI, body mass index; CI, confidence interval; FSH, follicle stimulating hormone; HRT, hormone replacement therapy; OR, odds ratio.
